# Bacterial Pleckstrin Homology Domains: A Prokaryotic Origin for the PH Domain

**DOI:** 10.1016/j.jmb.2009.11.006

**Published:** 2010-02-12

**Authors:** Qingping Xu, Alex Bateman, Robert D. Finn, Polat Abdubek, Tamara Astakhova, Herbert L. Axelrod, Constantina Bakolitsa, Dennis Carlton, Connie Chen, Hsiu-Ju Chiu, Michelle Chiu, Thomas Clayton, Debanu Das, Marc C. Deller, Lian Duan, Kyle Ellrott, Dustin Ernst, Carol L. Farr, Julie Feuerhelm, Joanna C. Grant, Anna Grzechnik, Gye Won Han, Lukasz Jaroszewski, Kevin K. Jin, Heath E. Klock, Mark W. Knuth, Piotr Kozbial, S. Sri Krishna, Abhinav Kumar, David Marciano, Daniel McMullan, Mitchell D. Miller, Andrew T. Morse, Edward Nigoghossian, Amanda Nopakun, Linda Okach, Christina Puckett, Ron Reyes, Christopher L. Rife, Natasha Sefcovic, Henry J. Tien, Christine B. Trame, Henry van den Bedem, Dana Weekes, Tiffany Wooten, Keith O. Hodgson, John Wooley, Marc-André Elsliger, Ashley M. Deacon, Adam Godzik, Scott A. Lesley, Ian A. Wilson

**Affiliations:** 1 Joint Center for Structural Genomics, http://www.jcsg.org; 2 Stanford Synchrotron Radiation Lightsource, SLAC National Accelerator Laboratory, Menlo Park, CA 94025, USA; 3 The Wellcome Sanger Institute, Wellcome Trust Genome Campus, Hinxton, Cambridge CB10 1SA, UK; 4 Protein Sciences Department, Genomics Institute of the Novartis Research Foundation, San Diego, CA 92121, USA; 5 Center for Research in Biological Systems, University of California, San Diego, La Jolla, CA 92093, USA; 6 Program on Bioinformatics and Systems Biology, Burnham Institute for Medical Research, La Jolla, CA 92037, USA; 7 Department of Molecular Biology, The Scripps Research Institute, La Jolla, CA 92037, USA; 8 Photon Science, SLAC National Accelerator Laboratory, Menlo Park, CA 94025, USA

**Keywords:** PH, Pleckstrin homology, PH*b*, bacterial PH domain, PTB, phosphotyrosine binding, VPS36, vacuolar protein sorting protein 36, DUF1696, domain of unknown function family 1696, JCSG, Joint Center for Structural Genomics, MAD, multiwavelength anomalous diffraction, PEG, polyethylene glycol, asu, asymmetric unit, PDB, Protein Data Bank, PIPE, Polymerase Incomplete Primer Extension, TEV, tobacco etch virus, TCEP, tris(2-carboxyethyl)phosphine–HCl, SSRL, Stanford Synchrotron Radiation Lightsource, ALS, Advanced Light Source, Pleckstrin homology (PH) domain, bacterial PH domain (PH*b*), higher-order symmetry, protein assembly, protein evolution

## Abstract

Pleckstrin homology (PH) domains have been identified only in eukaryotic proteins to date. We have determined crystal structures for three members of an uncharacterized protein family (Pfam PF08000), which provide compelling evidence for the existence of PH-like domains in bacteria (PH*b*). The first two structures contain a single PH*b* domain that forms a dome-shaped, oligomeric ring with C_5_ symmetry. The third structure has an additional helical hairpin attached at the C-terminus and forms a similar but much larger ring with C_12_ symmetry. Thus, both molecular assemblies exhibit rare, higher-order, cyclic symmetry but preserve a similar arrangement of their PH*b* domains, which gives rise to a conserved hydrophilic surface at the intersection of the β-strands of adjacent protomers that likely mediates protein–protein interactions. As a result of these structures, additional families of PH*b* domains were identified, suggesting that PH domains are much more widespread than originally anticipated. Thus, rather than being a eukaryotic innovation, the PH domain superfamily appears to have existed before prokaryotes and eukaryotes diverged.

## Introduction

The Pleckstrin homology (PH) domain is a common protein module in eukaryotes found in proteins with a wide range of functions, involved in intracellular signaling and cytoskeletal organization.^1,2^ The PH domain consists of a seven-stranded β-sandwich, which forms a pair of perpendicular β-sheets capped by a C-terminal amphipathic α-helix.^3–6^ PH domains are best known for binding phosphatidylinositol lipids and targeting proteins to the membrane.^7^ The PH domain-like fold was also identified in proteins that had very low sequence similarities to conventional PH domains. According to the SCOP database,^8^ they include phosphotyrosine binding (PTB) domain, FERM (band 4.1, ezrin, radixin, moesin homology) domain, Ran-binding domain, Enabled/VASP homology 1 domain, the GRAM domain of myotubularin, Dcp1 (decapping protein involved in mRNA degradation Dcp1), the GLUE domain of VPS36 (vacuolar protein sorting protein 36), the p62 subunit of TFIIH basal transcription factor complex, the POB3 subunit of the FACT complex, neurobeachin, and Necap1 (NECAP endocytosis associated 1). Although the overall topology of these PH-like domains is highly conserved, the loops connecting their core secondary structural elements are highly variable. PH-like domains, which define a superfamily of proteins with a PH domain fold (SCOP ID: 50729), are likely evolutionarily related to the PH domain. Proteins containing PH-like domains are essential in eukaryotes and PH-like domains are among the most abundant modules (fourth) in the human genome.^9^ As most characterized PH-like modules are involved in localizing proteins to specific cellular locations or protein partners, it was suggested that the PH-like module is a general targeting or “adaptor” domain.^10^

Despite the abundance of PH-like domains in eukaryotes, they had not been previously identified in bacteria. As part of our structural genomics effort in targeting novel protein families, we determined crystal structures of three bacterial proteins from a previously structurally uncharacterized protein family Pfam PF08000 [also known as domain of unknown function family 1696 (DUF1696)],^11^ which appears to be involved in the bacterial cell envelope stress response. These structures share a common domain that is surprisingly similar to the eukaryotic PH-like domains, thus providing the first direct evidence for the existence of PH-like domains in prokaryotes. We, therefore, suggest a much older origin for PH-like domains by illustrating that bacterial PH-like (PH*b*) domains are likely evolutionarily related to eukaryotic PH-like domains. The PH*b* proteins form oligomeric ring assemblies of different sizes and symmetries, providing an excellent example of how molecular architecture can evolve, where the general form of the molecular assembly is preserved, but its size is expandable. Furthermore, we show that several other uncharacterized protein families are likely to contain PH*b* domains. With more than 1000 prokaryotic homologs identified so far, it seems that this structural module is also widespread among bacteria and archaea.

## Results

### Sequence analysis of the bacterial PF08000 family

PF08000 defines a small protein family whose members are distributed in bacteria, bacteriophages, and archaea, with the majority in firmicutes and proteobacteria. As this family is of unknown function, as well as being structurally uncharacterized, it is also classified as DUF1696 in the Pfam database. One homolog has been identified in *Methanobrevibacter smithii*, an archaeal member of the human gut microbiota. Most members of this family have a sequence length of ∼ 125 residues (group 1), but a second group is longer (∼ 204 residues) with a C-terminal extension, and such sequences are detected only in Bacillaceae (e.g., *Bacillus subtilis*). Similarities in the core residues suggest that both groups share a common evolutionary origin, but group 2 likely has evolved more recently due to its narrower distribution in bacteria and its more restricted sequence diversity (∼ 50% identical). A third group of more distant homologs, containing the core sequence of the first group at their C-termini, is present in phages, such as *Lactococcus* phage bIL285 (ORF2, 181 residues) and *Staphylococcus* phage X2 (ORF15, 241 residues).

### Structure determination and quality of the models

To gain insights into this family of proteins, we determined crystal structures of three bacterial members (that represent groups 1 and 2) of the PF08000 family at the Joint Center for Structural Genomics (JCSG†) using its high-throughput structural genomics pipeline.^12^ The proteins from *Shewanella loihica* (*sl*), *Shewanella amazonensis* (*sa*), and *Exiguobacterium sibiricum* (*es*) were expressed in *Escherichia coli* as selenomethionine derivatives. As full-length (*sl*) and (*es*) failed to crystallize, different constructs with serial truncations of up to 16 residues at the N- and/or C-terminus, in steps of 4 residues, were screened in parallel. Only one N-terminal truncation construct of each protein, residues 9–124 for (*sl*) and 13–204 for (*es*), resulted in successful structure determination. Structure determination of full-length (*sa*) was enabled using reductive methylation of the protein prior to crystallization. All three structures were solved independently using the multiwavelength anomalous diffraction (MAD) phasing method.

**Fig. 1:**
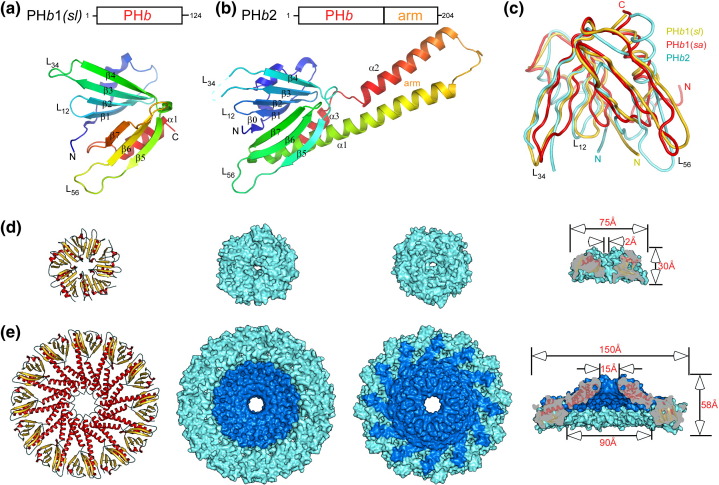
Structures of PH*b*1s and PH*b*2. (a) Ribbon diagram of PH*b*1(*sl*) monomer color coded from N-terminus (blue) to C-terminus (red). A schematic diagram of the domain architecture of PH*b*1(*sl*) is shown at the top. (b) Ribbon diagram of PH*b*2 monomer. (c) A structural superposition of the PH*b* domains of PH*b*1(*sl*) (gold), PH*b*1(*sa*) (red), and PH*b*2 (cyan). (d) PH*b*1(*sl*) pentamer shown in ribbons (bottom or base view) and surface representations (bottom, top and side views). (e) PH*b*2 dodecamer shown in same representation as PH*b*1. The PH*b* domain is colored cyan, as in PH*b*1. The PH*b*2 helical-hairpin arm attachment (residues 136–204) is colored blue.

**Fig. 2:**
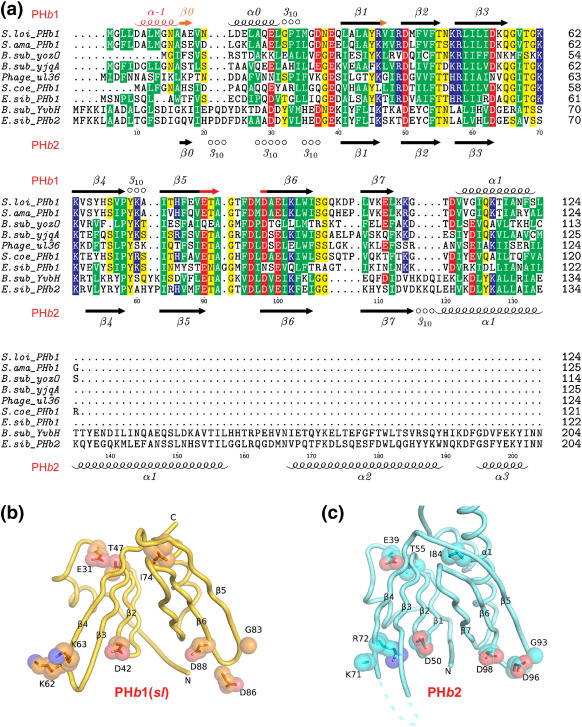
Sequence conservation of PH*b* homologs. (a) Sequence alignment of PH*b*s from *S. loihica*, *S. amazonensis*, *B. subtilis* (YozO, YjqA, and YvbH), *Lactococcus* phage ul36.k1, *Streptomyces coelicolor*, and *E. sibiricum*. The secondary-structure elements of the two PH*b*1s are shown at the top [red, unique to PH*b*1(*sa*); orange, unique to PH*b*1(*sl*); black, common]; secondary structure and sequence numbering of PH*b*2 are shown at the bottom. The conserved residues are highlighted (green, hydrophobic; yellow, polar; blue, basic; red, acidic). (b) Mapping of highly conserved residues onto the PH*b* domain of PH*b*1 with the side chains shown in ball and stick surrounded by their van der Waals surface. (c) Mapping of highly conserved residues onto the PH*b* domain of PH*b*2, color coded as in (b).

To our surprise, these structures had similarity to eukaryotic PH-like domains that we now define as bacterial PH domain-like proteins (Fig. 1) from group 1 (PH*b*1) {*S. loihica* [PH*b*1(*sl*), residues 9–124] and *S. amazonensis* [PH*b*1(*sa*), residues 1–125]} and from group 2 (PH*b*2) [*E. sibiricum* 255-15 (PH*b*2, residues 13–204)]. The two PH*b*1s are closely related (80% sequence identity); PH*b*1(*sa*) can be aligned to PH*b*1(*sl*) without gaps except for an extra glycine at its C-terminus (Fig. 2).

Both PH*b*1 structures were solved in orthorhombic space group *P*2_1_2_1_2_1_ at 2.0 Å resolution with one pentamer in the asymmetric unit (asu) [*R*_cryst_ = 18.6/*R*_free_ = 22.7 for PH*b*1(*sl*), Protein Data Bank (PDB) ID: 3dcx; *R*_cryst_ = 19.1/*R*_free_ = 23.3 for PH*b*1(*sa*), PDB ID: 3hsa]. PH*b*2 (PDB ID: 3b77) was solved in tetragonal space group *P*4 at 2.42 Å resolution with six molecules per asu (two 1/4 dodecamers) (*R*_cryst_ = 21.6/*R*_free_ = 25.4). The mean residual errors of the coordinates for PH*b*1(*sl*), PH*b*1(*sa*), and PH*b*2 were estimated at 0.16, 0.17, and 0.24 Å, respectively, by an *R*_free_-based diffraction-component precision index method.^13^ Analysis of the resulting models with MolProbity^14^ indicated good geometry with an all-atom clash score of 6.90, 0.4% rotamer outliers, and 98.0% of residues (no outliers) in the favored region of the Ramachandran plot for PH*b*1(*sl*) [NB: similar scores for PH*b*1(*sa*)] and an all-atom clash score of 6.82, 1.9% rotamer outliers, and 97.7% of residues in the favored region of the Ramachandran plot (five outliers in regions of poor density) for PH*b*2. The final model of PH*b*1(*sl*) contains 561 amino acid residues, 1 chloride ion, 7 (4*S*)-2-methyl-2,4-pentanediols, and 322 waters in the asu. The PH*b*1(*sa*) model consists of 585 protein residues, 2 polyethylene glycol (PEG) fragments, 6 glycerols, and 242 waters, and PH*b*2 has 1106 residues and 173 waters. Data collection, model, and refinement statistics are summarized in Table 1 (and Tables S1–S3).

### PH*b*1 and PH*b*2 monomers

PH*b*1 and PH*b*2 share a common domain that is structurally similar to eukaryotic PH domains (Fig. 1a–c), which we have defined as a PH*b* domain (PH domain in *bacteria*). PH*b*1(*sl*) (residues 9–124) has only a single PH*b* domain that consists of the canonical seven β-strands in two β-sheets (β1–β4 and β5–β7) and a C-terminal helix (α1) (Fig. 1a). It has an additional short β-strand β0 that connects to β1 with a large loop containing helix α0 that packs against the β1–β4 surface. PH*b*2 contains the PH*b* domain at its N-terminus (residue 14–136) and a helical-hairpin structure (∼ 68 residues) at the C-terminus (Fig. 1b) that extends out and returns as two α-helices (α1, α2) to the PH*b* domain. A short helix at the C-terminus (α3) docks between α1 and the β2–β3 (L_23_) loop and, as a result, links the other end of the hairpin to the PH*b* domain. This hairpin attachment is crucial for formation of the PH*b*2 homododecamer.

**Supplementary Fig. 1:**
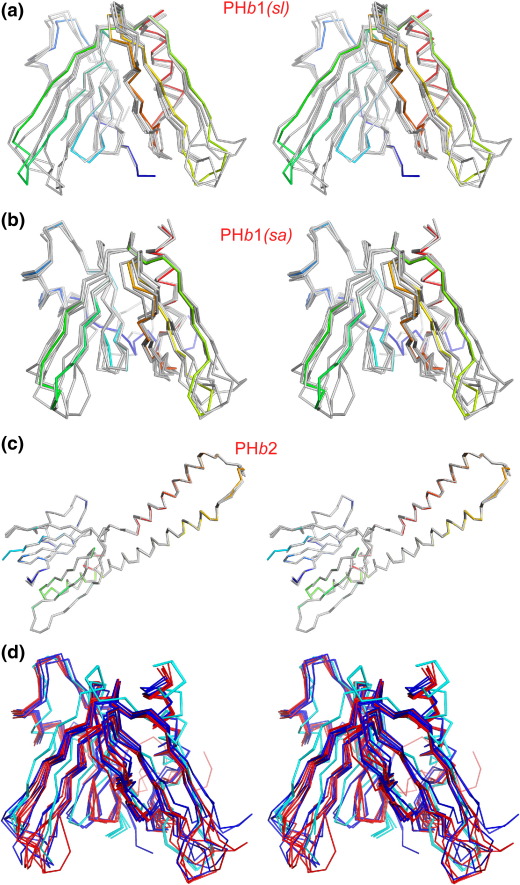


**Supplementary Fig. 2:**
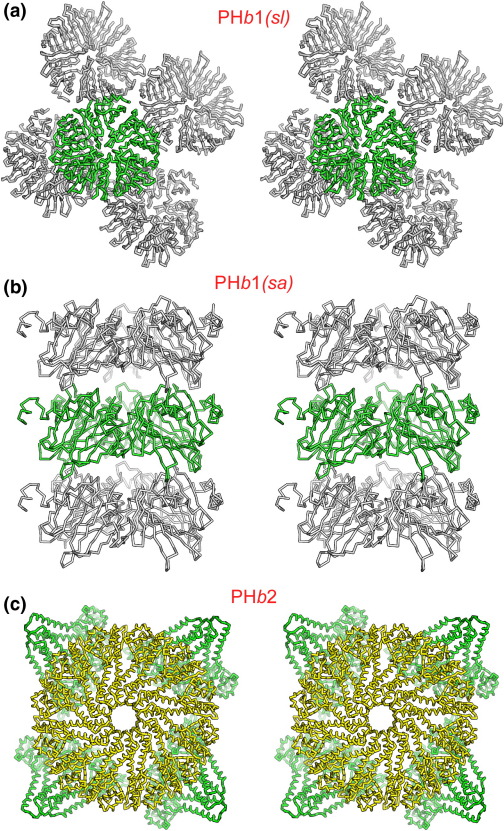


The overall shape of the PH*b* domain resembles a butterfly of two β-sheets with the β3–β4 loop (L_34_) and the β5–β6 loop (L_56_) at the tip of each wing. The PH*b* domains in two PH*b*1 orthologs contain a structurally conserved core, except for the N-termini (before α0), the L_34_ loop, and the L_56_ loop regions that are highly variable in conformation (Fig. S1). The 10 copies of the PH*b*1 monomers [from the two pentamers of PH*b*1(*sl*) and PH*b*1(*sa*)] can be superimposed with an average rmsd of 1.31 Å for 94 aligned C^α^ atoms; the rmsd values between different monomers are significantly higher if all variable regions were included (Tables S4–S6). The N-terminal residues, which were deleted in the PH*b*1(*sl*) construct, are ordered in two monomers (A and B) of PH*b*1(*sa*) due to crystal contacts and form a short helix (α-1, residues 5–11). The conformation of the N-terminal region differs significantly between PH*b*1(*sa*) and PH*b*1(*sl*) (Fig. 1c). PH*b*1(*sa*) does not contain the first strand (β0) found in PH*b*1(*sl*). These structural variations, which likely reflect differences in the contact environments in the crystals, demonstrate the inherent flexibility of the PH*b* domains (Fig. S2). On the other hand, the six independent protomers in the asu of PH*b*2 have similar packing environment and are essentially identical with each other in structure with an average rmsd of 0.27 Å for 180 aligned C^α^ atoms. The ordered L_56_ loops in PH*b*2 are also involved in crystal contacts, although to a lesser extent than PH*b*1(*sl*). The L_34_ loop of PH*b*2 is exposed to solvent and disordered. The PH*b* domains of PH*b*1 and PH*b*2 can be superimposed with an average rmsd of ∼ 3.0 Å for ∼ 110 aligned C^α^ atoms (Table S7). The main differences between the two PH*b* domains in PH*b*1(*sl*) and PH*b*2 are found in α0, the second β-sheet (β5–β7), α1, and the loop regions (Fig. 1c).

Mapping of conserved residues within the PH*b* family onto the structures of PH*b*1 and PH*b*2 shows high conservation of residues in strand β2, the L_34_ loop, and the L_56_ loop (Fig. 2). A highly conserved glutamate (Glu31 in PH*b*1 and Glu39 in PH*b*2) is buried inside the protein where its carboxyl side chain forms multiple hydrogen bonds with the main-chain amide (NH) of a conserved threonine (Thr47 in PH*b*1 and Thr55 in PH*b*2) and the side chain of an arginine (Arg50 in PH*b*1 and Arg76 in PH*b*2). These interactions, combined with the conserved hydrophobic core, are expected to be important for the structural integrity of PH*b* domains. Furthermore, Arg50 and Arg76 also participate in a water-mediated hydrogen-bond network at the oligomeric interfaces. The conserved residues that are clustered near the L_34_ and L_56_ loops are solvent exposed and are, thus, more likely to be of functional significance.

### Comparisons of PH*b* with eukaryotic PH-like domains

**Fig. 3:**
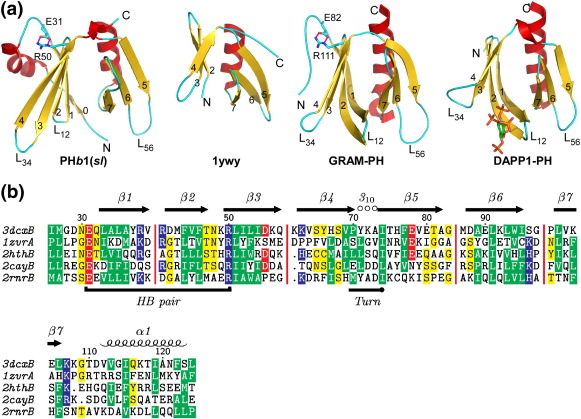
Comparisons of bacteria PH domains with eukaryotic PH domains. (a) Representative PH domains shown in same orientations: PH*b*1(*sl*) (PDB ID: 3dcx), uncharacterized protein PA2021 from *Pseudomonas aeruginosa* (PDB ID: 1ywy), the GRAM-PH domain myotubularin of (PDB ID: 1zvr), and the PH domain of DAPP1/PHISH complexed with inositol-(1,3,4,5)-tetrakisphosphate (PDB ID: 1fao). (b) Structure-based sequence alignment of PH*b* domain (PDB ID: 3dcx) with top hits of a DALI search: GLUE-PH domains (PDB ID: 2cay and 2hth), GRAM-PH (PDB ID: 1zvr), and TFIIH-PH (PDB ID: 2rnr). Red vertical bars indicate omitted gaps in the alignment.

DALI structural similarity searches,^15^ limited to the PH*b* domain from either PH*b*1 or PH*b*2, identified a large number of proteins from the eukaryotic PH superfamily. The top hits included proteins of diverse function, such as the GRAM-PH domain of myotubularin family phosphoinositide phosphatase MTMR2^16^ (PDB ID: 1zvr, *Z* = 9.9, rmsd = 2.3 Å for 94 aligned C^α^ atoms, 14% sequence identity), GLUE domains of VPS36 of human and *Saccharomyces cerevisiae*^17,18^ (PDB ID: 2hth, *Z* = 9.6, rmsd = 2.1 Å for 90 aligned C^α^ atoms, 16% id; PDB ID: 2cay, *Z* = 9.3, rmsd = 2.4 Å for 90 aligned C^α^ atoms, 13% id), and Dcp1^19^ (PDB ID: 2qkl, *Z* = 8.8, rmsd = 2.3 Å for 93 aligned C^α^ atoms). Three previously solved structures of uncharacterized bacterial proteins, two by the JCSG (PDB IDs: 2ra9 and 2re3) and one by the Northeast Structural Genomics Consortium (PA2021, PDB ID: 1ywy), also appear to contain a domain with PH fold but have one less β-strand (β1) and are structurally more distant. The closest eukaryotic protein in terms of structural similarity to PA2021 is the Ras-related protein RalA^20^ (PDB ID: 1zc3, *Z* = 3.4, rmsd = 2.5 Å for 59 aligned C^α^ atoms, 10% id). Structural comparisons of the PH*b*1 domain with PA2021 and representative eukaryotic PH-like domains are shown in Fig. 3. The conserved core consists of 7 β-strands (β1–β7) and a C-terminal α-helix (α1). Like eukaryotic PH-like domains, the β1–β4 sheet of PH*b*s packs perpendicularly with the β5–β7 sheet through hydrophobic contacts. An additional connection between the two β-sheets is mediated through the conserved C-terminal α-helix (α1). The most significant structural difference between PH*b* domains and canonical eukaryotic PH-like domains is the length and conformation of the β1–β2 hairpin. The longer hairpin of the eukaryotic PH-like domain curves towards the open edge of the β5–β7 sheet, such that β1 interacts with β7 to form an extended antiparallel β-sheet, thus producing a closed barrel. In contrast, the β1–β2 hairpin of PH*b*s, including the two-residue L_12_ loop, is shorter and does not interact with the β7 strand directly. The first ∼ 30 N-terminal residues of PH*b*s are not conserved across the PH-like superfamily of proteins. However, some proteins in the PH-like family do contain extra structural elements that are functionally equivalent for stabilization of the β1–β4 surface. For example, Dcp1 contains a helix that is equivalent to α0 of PH*b*1.

### Oligomeric rings of PH*b*

PH*b*1 and PH*b*2 form oligomeric rings with rarer, higher-order, cyclic symmetry.^21^ The structures and dimensions of the PH*b*1(*sl*) pentamer and the PH*b*2 dodecamer are illustrated in Fig. 1d and e. PH*b*1(*sl*) and PH*b*1(*sa*) form a disk with non-crystallographic C_5_ symmetry that is normally only encountered in viruses with icosahedral capsids. A hole in the center of the pentamer narrows from the top towards the base. The PH*b*1(*sl*) pentamer buries a total surface area of 9080 Å^2^ (34% of total surface area, 1816 Å^2^ per monomer). The PH*b*1(*sa*) pentamer is similar to that of PH*b*(*sl*); thus, we refer only to PH*b*1(*sl*) in the discussion hereafter. PH*b*2 is also a dome-shaped assembly, but with C_12_ cyclic symmetry that is generated by crystallographic C_4_ axis and a non-crystallographic C_3_ axis. The PH*b*2 assembly is significantly larger with an outer diameter of 150 Å, that is, twice that of PH*b*1(*sl*). The PH*b*2 dodecamer buries a total surface area of 46,700 Å^2^ or ∼ 50% of total surface area (3890 Å^2^ per monomer). Its central channel is also significant larger with a diameter of 90 Å at the base and 15 Å at the top (NB: the actual opening would likely be smaller if the Arg161 side chains around the top channel were ordered). The openings of both central channels are guarded by positively charged residues [Lys72 and Lys99 in PH*b*1(*sl*) and Arg161 in PH*b*2]. Both oligomeric states are consistent with size-exclusion chromatography experiments. Despite significant differences in the sizes of the two assemblies, the orientations of the PH*b* domains within the rings are highly similar. The base of each ring consists of repeating β-blades, while the top of each ring is primarily helical. If we superpose one subunit from the PH*b*1(*sl*) pentamer with one from the PH*b*2 dodecamer, then the average rotation angle that relates adjacent monomers from the two assemblies is 43° (standard deviation of 1.6°; range, 41–45°), which is very close to the expected value of 42° (360°/5 − 360°/12), if the deviation in rotation angle between monomers is uniform over the pentamer.

The oligomerization mode of the PH*b* domain resembles those of other intermolecular β-sheet forming assemblies of structurally different folds, for example, the Sm-like ribonucleoproteins (SCOP ID: 50182) and the TRAP-like superfamily of proteins (SCOP ID: 51219) in SCOP.^8^ These propeller-like assemblies also possess high-order cyclic symmetries and show variations of the number of subunits in the ring. GroES also has a dome-shaped architecture;^22^ however, the fold of the GroES monomer and the assembly details of the heptamer are different from those of PH*b*s.

### PH*b*1 pentamer

**Fig. 4:**
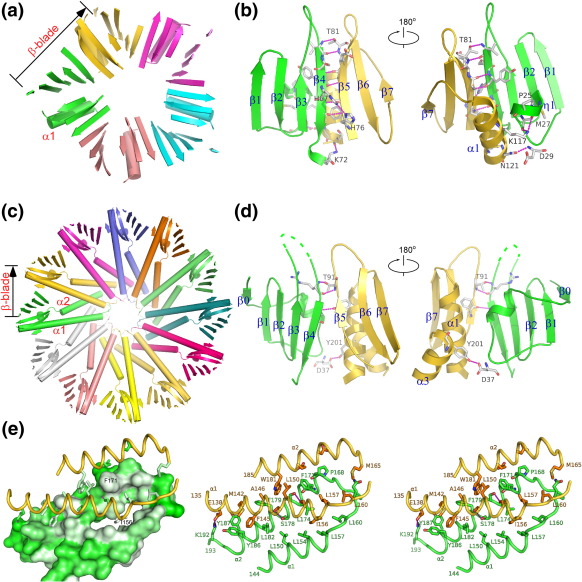
Dissection of PH*b* assembly interfaces. (a) Ribbon diagram showing the β-sheets and α1 of the PH*b*1(*sl*) pentamer. (b) Interface between two adjacent PH*b*1(*sl*) protomers (green and gold). Hydrogen bonds are shown as dashes (magenta). (c) Ribbon diagram showing the β-sheets and α1–α2 of the PH*b*2 dodecamer. (d) Interface between two adjacent PH*b*2 protomers (green and gold). (e) Stereo view of interactions between α1 and α2 hairpins of two adjacent protomers (right). A combined surface/ribbon representation of the same view is shown on the left. The surface is colored according to hydrophobicity, where greenish gray is the most hydrophobic.

The formation of the pentamer in PH*b*1(*sl*) is mediated primarily through hydrogen-bonding interactions (Fig. 4a and b). β4 and β5 of two adjacent protomers form an antiparallel β-sheet, consisting of five hydrogen bonds between their main-chain atoms. As a result, the β0–β4 and β5–β7 of the next protomer form an extended eight-stranded β-sheet, arranged like the blades of a propeller (β-blade) at the base of the pentamer. Additionally, His67 hydrogen bonds with His76 on the adjacent strand. The Thr81 hydroxyl group interacts with a main-chain amide. This conserved interaction (Thr91 in PH*b*2) helps to stabilize the β4 N-terminus with the β5 C-terminus of the next protomer and is likely important for PH*b* function since the proposed binding site is in close proximity (see below).

The second contact point in the pentamer involves α1 (residues 113–121) from one monomer and the 3_10_ helix region (residues 25–29) of the adjacent protomer. Lys117 adopts different conformations in different promoters and makes polar contact with different partners, such as the carbonyl groups of Met27 and Gly24 and the side chain of Asp29. Asn121Oδ1 forms another hydrogen bond with the main-chain amide of Asp29. Additionally, a water-mediated hydrogen-bonding network is observed for polar/charged residues buried in the interface (Arg50, Asn116, and Ser68). Overall, the PH*b*1(sl) pentamer interfaces lack specific, conserved, side-chain interactions, except for Thr81.

### Ring expansion in PH*b*2 through addition of a helical hairpin

The PH*b*2 α1 is significantly longer and forms a hairpin appendage with an additional helix α2 at the C-terminus (distance = 6.8 Å and Ω = − 159° to α1). The hairpin constitutes the primary mediator of the central core of the dodecameric PH*b*2 ring, mainly through hydrophobic interactions (Fig. 4c and e). Interactions between the α1 and α2 hairpins contribute to 63% (29,350 Å^2^) of the overall buried surface within the dodecamer. The packing of hairpins involves the docking of surface hydrophobic residues of α1 (around Ile156) and α2 (Val167, Phe171, and Phe179) into a hydrophobic pocket formed by the adjacent hairpin of the neighboring protomer (Fig. 4e). Helix α2 (near Ser175-Ser178) intersects with α1 of the next protomer (near Ser149-Ser153), with an inter-helix distance of 7.9 Å and an angle (Ω) of 134°. The four serines above are strategically located since their small side chain permits the helices to pack closely together and also form hydrogen bonds with each other, thus further stabilizing the interface.

Despite significant difference in the size of the oligomers, the arrangement of the PH*b* domains in the PH*b*2 dodecamer is remarkably similar to that of the PH*b*1 pentamer (Fig. 4c and d). The intersubunit eight-stranded β-sheets, located on the periphery of the ring, are arranged in a similar manner with respect to β4 and β5 of the two neighboring protomers. However, only two main-chain hydrogen bonds [compared to five in PH*b*1(*sl*)] are formed between the two strands due to the separation at the N-terminus of β4 and C-terminus of β5 to accommodate the packing of the α1–α2 hairpins. The second interface between PH*b* domains in PH*b*2 also resembles that of the PH*b*1 and involves contacts between the N-terminal 3_10_ helix of PH*b* (residues 31–37) and a section of helix α1 (residues 120–130) of two adjacent promoters. The interface involves van der Waals contacts (33% of nonpolar residues) but no hydrogen bonds. A region of helix α3 (residues 200–201), which is unique to PH*b*2, also contributes to these interactions (Tyr201/Asp37).

An additional structural adaption by the PH*b* domain of PH*b*2 was observed, presumably to facilitate formation of the dodecamer ring (Fig. 1c). The retraction of the β3–β4 and β5–β6 hairpin wings and the N-terminal inter-protomer contact region (residues 31–37) towards the center reduces the overall width of the PH*b* domain. A small change in the orientation of α1 towards the C-terminus widens the gap between α1 and L_23_ and allows docking of α3. PH*b*2 thus represents a fascinating example of protein evolution by which a new molecular architecture is derived from a simple modification of a basic module.

### Similarity of PH*b* oligomerization to protein recognition by eukaryotic PHs

**Fig. 5:**
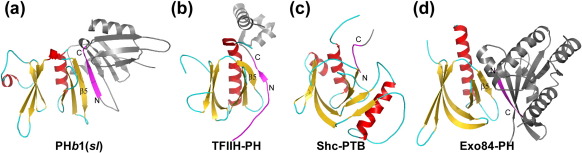
The oligomerization interface of PH*b* overlaps with a common protein interaction site of eukaryotic PH domains. The superimposed PH domains are shown in yellow/red/cyan. The protein/peptide partners are shown in gray with the interface elements highlighted in magenta. (a) PH*b*1(*sl*) dimer (PDB ID: 3dcx). (b) TFIIH (PH) complexed with TFIIE (PDB ID: 2rnr). (c) Shc PTB domain complexed with a phosphor-peptide (PDB ID: 1shc). (d) Exo84 PH domain complexed with RalA (PDB ID: 1zc3).

The oligomerization of PH*b*s involves an extension of the β-sheets at the open edge of β4 or β5, with additional contributions from the C-terminal α-helix (α1) and an N-terminal region containing a 3_10_ helix. Interestingly, this mode of protein–protein interaction, involving the open edge of the β5 strand, is commonly observed in eukaryotic PH-like domains (Fig. 5). In PTB domains, peptide ligands are bound as an antiparallel, pseudo-β-sheet with extensive contacts with the β5 strand and the C-terminal α-helix.^23^ Radixin utilizes the same interface, such that a β-strand binds the shallow groove between β5 and α1.^24,25^ Corresponding to the C-terminal β-turn NPxY motif of the PTB peptides, a conserved structural motif (^70^PYxxI^74^) exists at the C-terminus of β4 in PH*b*1 and adopts a similar main-chain conformation to PTB peptides, such as in Shc.^26^ However, the role of the NPxY motif tyrosine in PTB and PH*b*s is different; the tyrosine in the PTB peptides plays a significant role in PTB binding, while the tyrosine is buried inside PH*b*s and not involved in protein–protein interaction.

Another example of the β5–β7 β-sheet extension at β5 is observed in the recent structure of the TFIIH p62 subunit (PH domain) in complex with the C-terminal acidic domain of the general transcription factor TFIIE.^27^ Other modes of utilization of the β5 edge for protein–protein interaction were also observed. For example, Ral-binding domain of Exo84 utilizes the β5 edge for Ral interaction with the peptide from Ral adopting a parallel conformation.^20^ The interaction of the GLUE domain of VPS36 with ubiquitin does not directly involve a β-sheet type of interaction, but ubiquitin also occupies the β5 edge of the GLUE domain.^17^ Thus, the PH*b* oligomerization interface overlaps with a common site of protein–protein interaction in eukaryotic PH domains. Furthermore, the mode of PH*b* oligomerization through β-sheet extension is similar to the mode of protein recognition by eukaryotic PH domains.

### A conserved binding site

**Fig. 6:**
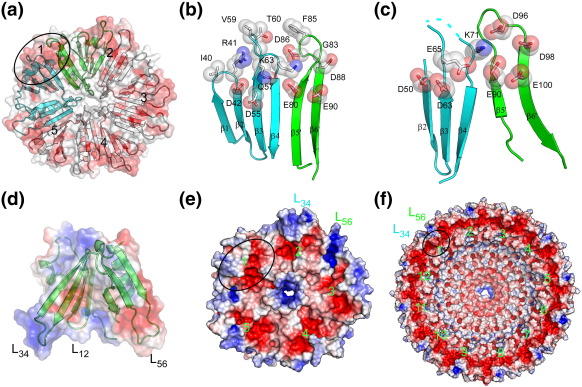
Potential binding sites of PH*b*s. (a) PH*b*1(sl) pentamer surface colored by sequence conservation (red, conserved; white, non-conserved). (b) Detailed view of the highly conserved residues and surface outlined in (a). (c) Similar conserved site on PH*b*2. (d–f) Electrostatic potentials of PH*b*1(*sl*) monomer, pentamer, and PH*b*2 dodecamer mapped to their protein surfaces. The color is scaled from − 5 to 5 kT/e for oligomers and from − 3 to 3 kT/e for the monomer (blue, positive electrostatic potential; red, negative electrostatic potential). Predicted binding sites are labeled 1–5 and 1–12, and one site is circled on each assembly.

Residues that are important for the structural integrity and function of a protein family are often under strong evolutionary constraints. Thus, we should be able to predict the functional sites of PH*b* assemblies by analyzing the sequence conservation patterns in the context of the protein structure, particularly for clustering of conserved residues on the protein surface. Aside from the few buried, charged residues discussed above, all conserved surface residues cluster to a single location on each β-blade at the base of the dome (Fig. 6a). Each site is formed by a contribution from two adjacent protomers, thus clearly indicating the physiological relevance of the oligomers. In PH*b*1(*sl*), these residues include Asp42 from β2, Asp55 and Gln57 from β3, and Lys63 from β4, as well as residues from the adjacent protomer: Glu80 from β5 and Asp86, Asp88, and Glu90 from β6 (Fig. 6b). The residues are mostly acidic, except for Lys63 in the middle. Residues from L_12_, L_34_, and L_56_ loops contribute to the perimeter of this site (e.g., Ile40, Arg41, Val59, Thr60, and Phe85). The surface is stabilized by residues (Asp40, Arg41, Thr81, Asp86, and Asp88) that form hydrogen bonds with adjacent structural elements. These PH*b*1 residues are also highly conserved in PH*b*2. Thus, PH*b*2 also contains a similar site due to the similarity in the arrangement of its PH*b* domain in the oligomer (Fig. 6c). The conformational flexibility of L_34_ and L_56_ loops observed in the crystal structures could be functionally relevant as they are located near the conserved binding sites.

The combination of structural elements from two adjacent PH domains, which form a single binding site, seems to be a novel feature unique to PH*b* domains, as no similar arrangements are observed in eukaryotic PHs. In fact, the eukaryotic PH domains often exist as a single module in a large protein, and most do not appear to be functionally dependent on oligomerization via their PH domains. One exception is mouse α1-syntrophin, which self-associates through its N-terminal PH domain.^28^

**Supplementary Fig. 3:**
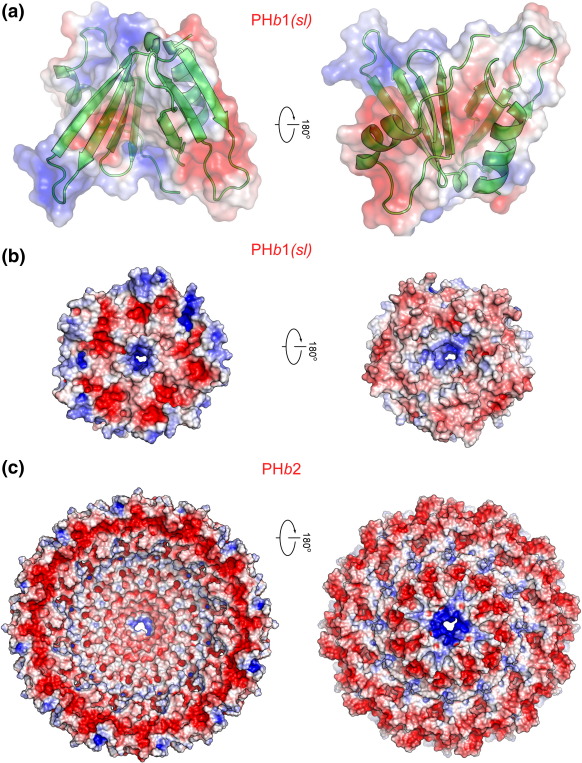


The bipolar electrostatic potential distribution (positive and negative potential partitioned between two ends of a molecule) displayed by many PH domains characterized to date is believed to facilitate their interaction with the membrane.^4,6^ The PH*b* domains are mostly electronegative except for a cluster of conserved positively charged residues on the top surface near L_34_ (Fig. 6d; Fig. S3). Thus, PH*b* domains do not display strong bipolarity. The oligomers of PH*b*1 and PH*b*2 are also electronegative with electropositive islands located at the perimeter of the dome (Fig. 6e and f). As the conserved binding sites of PH*b*s consist of mostly acidic residues, decorated with a single basic residue, electrostatic interaction (charge complementarity) is likely relevant for the function of PH*b*s.

## Discussion

### Evolution of PH domains

PH-like domains are ubiquitous in eukaryotes. The current SMART database (v6)^29^ has cataloged ∼ 15,000 proteins (SM00233) with one or more PH domains. Thus, it is surprising that no PH domains of prokaryotic origin have been previously reported. As PH domains are well known to be highly divergent, Ponting *et al.* noted that, if bacterial homologs were to exist, they would likely be undetectable by the sequence methods of the time.^30^ With the knowledge that PH-like domains do, indeed, exist in prokaryotes based on the PH*b*1 and PH*b*2 structures reported here, we used sequences from the PF08000 family to iteratively identify other potential homologs. Profile-based sequence similarity search methods implemented in PSI-BLAST^31^ identified proteins containing a GRAM domain, suggesting a possible evolutionary relationship between PH*b*s and eukaryotic PH domains. Another profile-based fold recognition method, FFAS, suggested that PH*b* domains were likely related to the GLUE domain (97% confidence).^32^ However, even with improved methods for detecting remote sequence homology in recent years, the probability scores given by these methods were relatively low. Thus, we further examined the conserved structure features in order to more rigorously establish whether any evolutionary relationship exists between PH*b* domains and eukaryotic PH domains. In the absence of strong sequence similarity, remote relatives of a highly divergent superfamily can be detected by analyzing unique structural motifs that are important for either fold or function.^33^

The PH*b* secondary-structure elements are highly related to those of eukaryotic PH-like domains, with similar highly conserved buried hydrophobic residues (Fig. 3b). Besides similarities in the overall fold, we identified several specific structural features that are important to PH*b* domains, which are also preserved in eukaryotic PH domains. The linker between β4 and β5 in PH*b* domains consists of a one-turn 3_10_ helix followed by a highly conserved isoleucine (consensus sequence ^70^PYxxI^74^ in PH*b*1, Fig. 2a). The side chains of Ile74 and Tyr71 point towards the buried hydrophobic cavity consisting of Leu51, Phe77, Leu93, and Ile119. Two hydrogen bonds are observed between main-chain atoms (Tyr71O-Ile74N and Pro70O-Ala73N in PH*b*1) in the 3_10_ helix preceding Ile74 in PH*b*1. This region is located at a strategic location where the first β-sheet (β1–β4), second β-sheet (β5–β7), and the C-terminal helix α1 pack together and is likely to be important for correct folding of the domain. This structural feature is widely conserved in the PH-like superfamily [e.g., GRAM-PH (PDB ID: 1zvr), GLUE domain (PDB ID: 2cay), and Bruton's tyrosine kinase (PDB ID: 1btk)], supporting its importance for the PH-like fold. Another conserved structural feature of PH*b*1 domains is a highly conserved, mostly buried salt bridge (Glu31-Arg50 in PH*b*1, Fig. 3a). Glu31 is located on the loop before β1 and Arg50 at the beginning of β3. Additionally, Arg50 also connects to β4 and the first 3_10_ helix through hydrogen bonds to Ser68Oγ and Ile26O. As a result, these interactions link multiple structural elements and likely contribute to structural stability. This ion pair is conserved in eukaryotic PH domains that are the closest structural homologs of PH*b*s, such as the GRAM-PH domain, GLUE domains, and TFIIH (Fig. 3b). As these key structural features are less likely to have been independently evolved, we suggest that bacterial and eukaryotic PH domains have diverged from a common ancestral fold. The fact that these domains use the same strategy in mediating protein–protein interactions also provides further clues for an evolutionary relationship.

The sequence diversity of PH-like domains could reflect their functional diversity. The binding sites of PH domains are generally functionally dependent and not restricted to a single location.^10^ However, with the expansion of our knowledge of PH-like domains, a few functional “hot spots” begin to emerge. One such site is the extended β1–β2 hairpin, which often is required for binding phospholipids. As the β-hairpin in PH*b* domains is shorter, we expect that they do not utilize this site for functional purposes. Interestingly, eukaryotic PH domains all have longer curved hairpins, even in proteins that do not appear to utilize that site. Thus, the β1–β2 hairpin extension is likely a eukaryotic-specific, structural adaption that provides new functionality. Further evidence of structural adaption of the β1–β2 hairpin is provided by PTB domains that contain an additional helix insertion. A second hot spot in PH-like domains is the open edge of β5, which is frequently used for mediating protein interaction, and it is this site that PH*b* domains utilize for oligomerization.

### Unifying uncharacterized protein families: DUF1696 (PF08000), DUF1200, DUF304, and DUF2244

To see whether we could expand the assignment of PH*b* domains to other families, we looked for relationships of protein families in PFAM using the SCOOP software, which uses a profile-based method to detect distant relationships between protein families.^34^ The highest-scoring family to DUF1696 is DUF304 with a score of 15.7. Although this score would normally not be considered significant, it was notable that the second best match was to the GRAM domain (score, 7.9) and the third best match was to Vps36_ESCRT-II (score, 6.9), whose structural similarities to PH*b*s are now confirmed from this work. SCOOP also identifies DUF304 as being related to DUF1200 with a highly significant score of 82.2. DUF2244 was the second highest-scoring match (score, 17.3) and DUF1696 was the third highest (score, 15.7). These analyses suggest that DUF2244 also possibly belongs to the PH*b* domain family. Based on the known PH*b* structures, we noted that the current Pfam definition of DUF2244 includes two N-terminal transmembrane helices that anchor this family to the membrane, much like DUF1200. Interestingly, alignments of each of these families suggest that they lack the β1 strand, similar to PA2021, and raise the interesting possibility that PH*b* domains lacking a β1 strand could represent a more simplified, ancestral-like structure (Fig. S4).

Apart from DUF1696, each of the DUF families that we identify as containing a PH*b* domain are potential transmembrane proteins where the PH*b* domain would be located intracellularly as predicted with the Phobius software.^35^ The challenge in solving the structure of membrane proteins is well known and perhaps explains why no PH*b* domains have been characterized previously. Given the potential for PH*b* domains to form oligomeric rings, we hypothesize that these proteins may act as membrane-bound transporters or pores with the PH*b* domains mediating oligomerization.

### Functions of PH*b* domains in the PF08000 family

As discussed above, a conserved hydrophilic site near the rim of the dome is likely to be of functional importance for both PH*b*1 and PH*b*2. The primary difference in their assembly is the size and symmetry of the ring structure; nevertheless, we expect that PH*b*1 and PH*b*2 may have similar functions at the molecular level. The physiological functions of PH*b* domains are currently not understood. Genome-wide studies have shown that the three paralogs of PH*b* in *B. subtilis* (YjqA, YozO, and YvbH) are expressed, indicating that PH*b*s are functional in bacteria.^36–38^ YozO of *B. subtilis* is induced under antibiotics, heat shock, and alkaline shock, suggesting a role in cell stress responses.^39–42^ YozO is under control of sigma factor σ^W^, a member of extracytoplasmic function (ECF) σ factors that often control functions associated with the cell surface or transport. σ^W^ likely plays a role in defending the cell against antimicrobial agents.^43^ The involvement of the YozO homolog in cell envelope stress response is also confirmed in *Bacillus licheniformis*.^44^ Another possible clue for PH*b* function may be provided by the presence of PH*b*1 in the lysogenic modules of bacteriophages, such as *Lactococcus* bacteriophage ul36 (ORF124)^45^ and Min1 phage from the nematode pathogen *Microbacterium nematophilum* (ORF77).^46^ Therefore, PH*b*s may also play a role in the phage life cycle.

YvbH of *B. subtilis* is found in the sub-membrane fraction and was predicted as a peripheral protein.^38^ PH*b*1 homologs were also identified in the insoluble sub-proteome of other bacteria, such as *Streptomyces coelicolor*^47^ (SCO3793, Swiss-Prot: Q9F325) and *Oceanobacillus iheyensis*^48^ (Swiss-Prot: Q8ELK9), an alkaliphilic and halotolerant deep-sea bacterium. These results raise the possibility that the activity of PH*b* domains may be associated with the membrane. However, the structures of PH*b*s do not seem to support a direct interaction, since the electronegative conserved binding sites of the PH*b* oligomers are not suitable for interacting with a negatively charged bacterial membrane. Furthermore, the PH*b* domain also lacks the actual site and electropositive surface corresponding to the eukaryotic lipid binding site. Instead, we predict that the PH*b*s likely interact with other proteins through the conserved binding site at their base. A eukaryotic example, which utilizes a similar location for protein interaction, is the TFIIE/TFIIH complex where the β5–β6 hairpin region of TFIIH is involved in the interaction with an extended peptide from TFIIE (Fig. 5). From the molecular architecture point of view, the PH*b*1 and PH*b*2 assemblies are similar to “caps” or “domes”. Thus, it is tempting to speculate that the PH*b* domain is likely an end piece of a molecular assembly, such as a membrane-associated protein complex (where different sizes of PH*b* domains were evolved to complement different sizes of channels/chambers). It may also be significant that these oligomers are assembled from higher-order cyclic symmetries that are rare in proteins analyzed to date,^21^ which may well provide some clues as to function. Further experimental studies are clearly needed for a more complete understanding of the biological function of PH*b* domains and assemblies, but the identification here of PH*b* domains provides fascinating new insights into their involvement in all kingdoms of life.

## Materials and Methods

### Protein production

The same protocol was used for the cloning, expression, and production of PH*b*1(*sl*) (Swiss-Prot: A3QB43), PH*b*1(*sa*) (Swiss-Prot: A1S3D0), and PH*b*2 (Swiss-Prot: Q41E03). Clones were generated using the Polymerase Incomplete Primer Extension (PIPE) cloning method.^49^ The gene encoding the targeted protein was amplified by polymerase chain reaction (PCR) from genomic DNA of the host bacteria (*Shewanella* sp. PV-4, *S. amazonensis* SB2B, and *Exiguobacterium* sp. 255-15) using PfuTurbo DNA polymerase (Stratagene) and I-PIPE (Insert) primers that included sequences for the predicted 5′ and 3′ ends. The expression vector pSpeedET, which encodes an amino-terminal tobacco etch virus (TEV) protease-cleavable expression and purification tag (MGSDKIHHHHHHENLYFQG), was PCR amplified with V-PIPE (Vector) primers. V-PIPE and I-PIPE PCR products were mixed to anneal the amplified DNA fragments together. *E. coli* GeneHogs (Invitrogen) competent cells were transformed with the V-PIPE/I-PIPE mixture and dispensed on selective LB-agar plates. The cloning junctions were confirmed by DNA sequencing. Using the PIPE method, the section of the gene encoding residues Met1-Met8 was deleted for PH*b*1(*sl*) (Met1-Phe12 was deleted for PH*b*2). Expression was performed in a selenomethionine-containing medium. At the end of fermentation, lysozyme was added to the culture to a final concentration of 250 μg/ml, and the cells were harvested and frozen. After one freeze/thaw cycle, the cells were homogenized in lysis buffer [50 mM Hepes, pH 8.0, 50 mM NaCl, 10 mM imidazole, and 1 mM tris(2-carboxyethyl)phosphine–HCl (TCEP)] and the lysate was clarified by centrifugation at 32,500*g* for 30 min. The soluble fraction was passed over nickel-chelating resin (GE Healthcare) pre-equilibrated with lysis buffer, the resin was washed with wash buffer [50 mM Hepes, pH 8.0, 300 mM NaCl, 40 mM imidazole, 10% (v/v) glycerol, and 1 mM TCEP], and the protein was eluted with elution buffer [20 mM Hepes, pH 8.0, 300 mM imidazole, 10% (v/v) glycerol, and 1 mM TCEP]. The eluate was buffer exchanged with TEV buffer (20 mM Hepes, pH 8.0, 200 mM NaCl, 40 mM imidazole, and 1 mM TCEP) using a PD-10 column (GE Healthcare) and incubated with 1 mg of TEV protease per 15 mg of eluted protein. The protease-treated eluate was passed over nickel-chelating resin (GE Healthcare) pre-equilibrated with Hepes crystallization buffer (20 mM Hepes, pH 8.0, 200 mM NaCl, 40 mM imidazole, and 1 mM TCEP), and the resin was washed with the same buffer. The flow-through and wash fractions were combined and concentrated for crystallization trials by centrifugal ultrafiltration (Millipore). For PH*b*1(*sa*), lysines were reductively methylated by adding 40 μl 0.98 M dimethylaminoborane and 80 μl 3.26% by weight formaldehyde, per milliliter of protein, for 2 h in the presence of crystallization buffer at 4 °C.^50^ Methylation reagents were subsequently removed using a PD-10 column.

### Crystallization of PH*b*1(*sl*)

PH*b*1(*sl*), concentrated to 13.3 mg/ml, was crystallized by mixing 100 nl protein with 100 nl crystallization solution in a sitting drop above a 50-μl reservoir volume using the nanodroplet vapor diffusion method^51^ with standard JCSG robotic crystallization protocols.^12^ The crystallization reagent consisted of 37% (v/v) 2-methyl-2,4-pentanediol, 0.15 M sodium chloride, and 0.1 M Hepes, pH 6.83. No additional cryoprotectant was added to the rod-shaped crystal (dimensions: ∼ 200 μm × 60 μm × 40 μm) grown at 277 K.

### Crystallization of PH*b*1(*sa*)

PH*b*1(*sa*) crystals were obtained in multiple conditions. The crystal used for refinement was obtained using a precipitating reagent consisting of 10% glycerol, 5% PEG 3000, 20% PEG 400, and 0.1 M Hepes, pH 7.3. (4*S*)-2-Methyl-2,4-pentanediol was added to the crystal as a cryoprotectant to a final concentration of 10% (v/v). The crystallization reagent yielding the crystal used for the second MAD data set consisted of 5% PEG 3000, 22% PEG 400, 10% glycerol, and 0.1 M Hepes, pH 7.5. Glycerol was added to the crystal as a cryoprotectant to a final concentration of 15% (v/v). Both cubic-shaped crystals (dimensions: ∼ 30 μm × 20 μm × 20 μm) were harvested after 50 days at 293 K. The protein concentration was 19 mg/ml.

### Crystallization of PH*b*2

The crystallization reagent consisted of 10% (w/v) PEG 6000 and 0.1 M *N*,*N*-bis(2-hydroxyethyl)glycine, pH 9.0. The diamond-shaped crystal (dimensions: ∼ 40 μm × 40 μm × 40 μm) was grown at 277 K. Ethylene glycol was added to the crystal as a cryoprotectant to a final concentration of 10% (v/v). The protein concentration was 13.6 mg/ml.

### Diffraction screening and oligomeric state determination

In order to identify the crystals with the best possible diffraction, we screened all harvestable crystal hits for diffraction using the Stanford Automated Mounting system^52^ at the Stanford Synchrotron Radiation Lightsource (SSRL, Menlo Park, CA). The molecular weight and oligomeric states of PH*b*1(*sl*) and PH*b*2 were determined using a 1 × 30 cm Superdex 200 column (GE Healthcare) in combination with static light scattering (Wyatt Technology). The mobile phase consisted of 20 mM Tris, pH 8.0, 150 mM NaCl, and 0.02% (w/v) sodium azide.

### Data collection, structure solution, and refinement

MAD data were collected at the Advanced Light Source (ALS, Berkeley, CA) beamline 8.2.2 (*S. loihica* PH*b*1) and SSRL beamlines 9-2 (*S. amazonensis* PH*b*1) and 11-1 (PH*b*2). Data were collected at wavelengths corresponding to the inflection, high-energy remote, and peak of a selenium MAD experiment at 100 K using ADSC Q315 [PH*b*1(*sl*)] and MarCCD325 [PH*b*2 and PH*b*1(*sa*)] detectors. Data processing, structure solution, and refinement were carried out independently for each of the three proteins using the following protocol. The MAD data were integrated and reduced using XDS and then scaled with the program XSCALE.^53^ Selenium sites were located with SHELXD.^54^ Phase refinement and automatic model building were performed using autoSHARP,^55^ RESOLVE,^56^ and wARP.^57^ For PH*b*1(*sa*), an additional MAD data set was collected using another crystal after initial higher-resolution SAD data did not provide enough phasing information. Structural solution was possible with combination of both data sets, which improved the accuracy of phases, especially at lower resolution. Model completion and refinement were performed with Coot^58^ and REFMAC5^59^ of the CCP4 suite.^60^ Tight non-crystallographic symmetry restraints were imposed for most regions of PH*b*2 except for flexible loop regions, while loose or no non-crystallographic symmetry restraints were used for PH*b*1s. Each monomer was defined as a TLS group. Experimental phases were used as restraints during refinement. Analysis of the stereochemical quality of the model was accomplished using MolProbity.^14^ All molecular graphics were prepared with PyMOL (DeLano Scientific) unless specifically stated otherwise. The alignment of multiple PH*b* domains and the average rmsd value were calculated using MATT.^61^ Pairwise structural comparisons in Tables S4–S6 were calculated with LSQKAB^62^ using a maximum common set of C^α^ atoms in both structures. Alignments of PH*b* domains in Table S7 were calculated using TMalign.^63^ Sequence alignments were rendered using TEXshade.^64^ Electrostatic potentials were calculated using APBS.^65^

Atomic coordinates and experimental structure factors for both PH*b*1s at 2.0 Å resolution and PH*b*2 at 2.42 Å resolution have been deposited in the PDB under accession codes 3dcx, 3hsa, and 3b77, respectively.

## Figures and Tables

**Table 1:** Summary of data collection and refinement statistics

	*S. loihica* PH*b*1	*S. amazonesis* PH*b*1	*E. sibiricum* PH*b*2
Structure (PDB ID)	(3dcx)	(3hsa)	(3b77)
Beamline	SSRL BL 11-1	SSRL 9-2	ALS 8.2.2
Space group	*P*2_1_2_1_2_1_	*P*2_1_2_1_2_1_	*P*4
Unit cell parameters (Å)			
*a*	61.07	33.23	150.99
*b*	75.32	129.49	150.99
*c*	139.40	138.73	76.20

*Data collection*	3dcx-infl	3hsa-peak	3b77-infl
Wavelength (Å)	0.9793	0.9792	0.9799
Resolution range (Å)	29.83–2.00	47.40–2.00	47.73–2.42
No. of observations	177,704	150,785	245,189
No. of unique reflections	43,831	41,702	65,459
Completeness (%)^a^	99.1 (97.9)	98.9 (98.6)	99.7 (99.8)
Mean *I*/σ (*I*)^a^	13.9 (2.3)	12.1 (2.6)	10.6 (1.9)
*R*_sym_ on *I* (%)^a^	6.7 (68.4)	8.8 (49.3)	8.1 (76.1)
Highest-resolution shell	2.11–2.00	2.10–2.0	2.55–2.42

*Model and refinement statistics*			
Resolution range (Å)	29.83–2.00	47.4–2.0	47.73–2.42
Cutoff criteria	|*F*| > 0	|*F*| > 0	|*F*| > 0
No. of reflections (total)	43,782	41,641	65,460
No. of reflections (test)	2200	2115	3326
Completeness (% total)	98.9	98.5	99.7
*R*_cryst_	18.4	19.1	21.4
*R*_free_	22.7	23.6	25.4

*Stereochemical parameters*			
Restraints (RMS observed)			
Bond length (Å)	0.014	0.015	0.015
Bond angle (°)	1.41	1.66	1.50
Average isotropic *B*-value^b^ (Å^2^)	35.4	27.5	65.6
ESU based on *R*_free_ (Å)	0.16	0.17	0.24
Chains/protein residues/atoms	5/561/4481	5/585/4675	6/1106/8993
Solvent molecules	330	250	173

ESU, estimated overall coordinate error. *R*_sym_ = ∑*_hkl_*∑*_i_*|*I_i_*(*hkl*) − 〈*I*(*hkl*)〉|/∑*_hkl_*∑*_i_I_i_*(*hkl*). *R*_cryst_ = ∑*_hkl_*||*F*_obs_| − |*F*_calc_||/∑*_hkl_*|*F*_obs_|, where *F*_calc_ and *F*_obs_ are the calculated and observed structure factor amplitudes, respectively. *R*_free_ = as for *R*_cryst_, but for 5.0% of the total reflections chosen at random and omitted from refinement.

a Statistics for the highest-resolution shell are in parentheses.

b This value represents the total *B* that includes TLS and residual *B* components.

## Footnotes

† http://www.jcsg.org
