# Supplementary material for: Bacterial Pleckstrin Homology Domains: A Prokaryotic Origin for the PH Domain
Source: J Mol Biol. 2010 Feb 12;396(1):31–46. doi: 10.1016/j.jmb.2009.11.006 (PMC2817789; doi:10.1016/j.jmb.2009.11.006)
Supplement: Supplementary Data [file mmc1.pdf]

Supplementary Data for "Bacterial Pleckstrin Homology Domains: a Prokaryotic Origin for the PH Domain"

FIGURE LEGENDS

Fig. S1. C^{α} superimposition of monomers in the asu PH*b*1(*sl*) (**a**), PH*b*1(*sa*) (**b**) and PH*b*2 (**c**). The first monomer in each panel was colored from blue to red from N-terminus to C-terminus. (**d**) Superimposition of all PHb domains (PH*b*1(*sl*): blue, PH*b*1(*sa*): red, PH*b*2: cyan), shown in stereo view.

Fig. S2. Crystal packing environments of PH*b*1(*sl*) (**a**), PH*b*1(*sa*) (**b**) and PH*b*2 (**c**).

Fig. S3. Electrostatic potentials of PH*b*1(*sl*) monomer (**a**), pentamer (**b**), and PH*b*2 dodecamer (**c**) mapped to their protein surfaces. The color is scaled from -5 to 5kT for oligomeric assemblies (blue, positive; red, negative electrostatic potential), and -3 to 3 kT for the PH*b*1(*sl*) monomer.

Fig. S4. Multiple sequence alignments of uncharacterized families that are likely to possess a PH*b*-like fold. Alignments show the UniProt accession number followed by the start and end coordinate of the domain in the sequence. For each alignment a secondary structure prediction is shown based on a JPred3 [1] prediction of the first sequence in the alignment (extended (E), helical (H) and other (-) types of secondary structure). Coloring of conservation has been carried out with the CHROMA software [2] (Conserved aliphatic residues are shown in bold black fonts with yellow background, residues with large side chains are shown in bold purple fonts with light yellow background, polar residues in blue text, and residue with small side chains are shown in green fonts). (**a**) Alignment of family DUF304. (**b**) Alignment of Family DUF1200. (**c**) Alignment of family DUF2244.

Supplementary References

1. Cole, C., Barber, J. D. & Barton, G. J. (2008). The Jpred 3 secondary structure prediction server. *Nucleic Acids Res.* **36**, W197-201.
2. Goodstadt, L. & Ponting, C. P. (2001). CHROMA: consensus-based colouring of multiple alignments for publication. *Bioinformatics,* **17**, 845-846.

Table S1. Data collection and phasing statistics for PH*b*1(*sl*)

Structure (PDB ID)	*S. loihica* PH*b*1 (3dcx)		
Beamline	SSRL BL 11-1		
Space group	$P2_12_12_1$		
Unit cell parameters	a=61.07 Å b=75.32 Å c=139.40 Å		
**Data collection**	3dcx-infl	3dcx-rem	3dcx-peak
Wavelength (Å)	0.9793	0.9184	0.9787
Resolution range (Å)	29.83-2.00	29.26-2.00	29.26-2.12
Number of observations	177,704	177,123	149,911
Number of unique reflections	43,831	43,614	37,021
Completeness(%) [a]	99.1(97.9)[a]	99.2(97.8)	99.3(98.2)
Mean I/σ (I) [a]	13.9(2.3)[a]	13.6(2.2)	14.7(2.7)
R_{sym} on I (%) [a]	6.7(68.4)[a]	7.4(71.0)	7.6(58.3)
Highest resolution shell	2.11-2.00	2.11-2.00	2.23-2.12
**Phasing**			
Resolution (Å)		30-2.0	
Number of Se sites		16	
Mean FOM		0.32	

[a] Statistics for the highest resolution shell in parentheses.

$R_{sym}=\Sigma_{hkl}\Sigma_i |I_i(hkl)-<I(hkl)>|/\Sigma_{hkl}\Sigma_i I_i(hkl)$

Table S2. Data collection and phasing statistics for PH*b*1(*sa*)

Structure (PDB ID)	*S. amazonesis* PH*b*1 (3hsa)			
Beamline	SSRL 9-2			
Space group	$P2_12_12_1$			
Unit cell parameters	a=33.23 Å b=129.49 Å c=138.73 Å	a=33.01 Å b=129.63 Å c=138.96 Å		
**Data collection**	3hsa-peak	3hsa-infl	3hsa-rem	3hsa-peak
Wavelength (Å)	0.9792	0.9793	0.9184	0.9792
Resolution range (Å)	47.4-2.0	43.6-2.7	47.4-2.7	43.6-2.7
Number of observations	150,785	61,112	61,189	61,111
Number of unique reflections	41,702	17,022	17,004	17,030
Completeness(%) [a]	98.9(98.6)	98.4(94.3)	98.4(94.3)	98.4(94.3)
Mean I/σ (I) [a]	12.1(2.6)	13.0(3.2)	13.6(3.3)	16.1(4.1)
R_{sym} on I (%) [a]	8.8 (49.3)	9.1(42.0)	8.6(41.6)	7.2(32.6)
Highest resolution shell	2.10-2.0	2.84-2.7	2.84-2.7	2.84-2.7
**Phasing**				
Resolution (Å)	47.4-2.7			
Number of Se sites	14			
Mean FOM	0.37			

[a] Statistics for the highest resolution shell in parentheses.

$R_{sym}=\Sigma_{hkl}\Sigma_i |I_i(hkl)-<I(hkl)>|/\Sigma_{hkl}\Sigma_i I_i(hkl)$

Table S3. Data collection and phasing statistics for PH*b*2

Structure (PDB ID)	PH*b*2 (3b77)		
Beamline	ALS 8.2.2		
Space group	P4		
Unit cell parameters	a=150.99 Å b=150.99 Å c=76.20 Å		
**Data collection**	3b77-infl	3b77-rem	3b77-peak
Wavelength (Å)	0.9799	1.000	0.9795
Resolution range (Å)	47.73-2.42	47.73-2.47	47.73-2.56
Number of observations	245,189	230,496	207,778
Number of unique reflections	65,459	61,521	55,605
Completeness(%) [a]	99.7(99.8)	99.7(99.9)	99.8(99.8)
Mean I/σ (I) [a]	10.6(1.9)	13.9(2.1)	10.4(2.0)
R_{sym} on I (%) [a]	8.1(76.1)	7.1(70.4)	9.2(73.2)
Highest resolution shell	2.55-2.42	2.60-2.47	2.70-2.56
**Phasing**			
Resolution (Å)	44-2.42		
Number of Se sites	20		
Mean FOM	0.27		

[a] Statistics for the highest resolution shell in parentheses.

$R_{sym}=\Sigma_{hkl}\Sigma_i |I_i(hkl)-\langle I(hkl)\rangle|/\Sigma_{hkl}\Sigma_i I_i(hkl)$

Table S4. Structural comparisons between monomers of PH*b*1(*sl*) pentamer

RMSD	3dcx-B	3dcx-C	3dcx-D	3dcx-E
3dcx-A	2.72 Å (113)[a]	1.50 (115)	2.24 (113)	1.70 (103)
3dcx-B		3.16 (113)	1.76 (113)	0.96 (103)
3dcx-C			2.55 (113)	1.80 (103)
3dcx-D				1.03 (103)
3dcx-E				

[a]Number of aligned C^{α} atoms in parenthesis.

Table S5. Structural comparisons between monomers of PH*b*1(*sa*) pentamer

RMSD	3hsa-B	3hsa-C	3hsa-D	3hsa-E
3hsa-A	3.62 (124)[a]	1.46 (112)	2.48 (113)	1.91 (111)
3hsa-B		2.03 (112)	2.24 (113)	1.94 (111)
3hsa-C			2.31 (112)	2.17 (111)
3hsa-D				2.00 (111)
3hsa-E				

[a]Number of aligned C^{α} atoms in parenthesis.

Table S6. Structural comparisons between monomers of PH*b*1(*sa*) and PH*b*1(*sl*)

RMSD	3dcx-A	3dcx-B	3dcx-C	3dcx-D	3dcx-E
3hsa-A	3.29 (116)[a]	2.15 (113)	3.62 (115)	2.20 (113)	1.38 (103)
3hsa-B	3.80 (116)	1.72 (113)	3.65 (115)	1.74 (113)	0.97 (103)
3hsa-C	2.69 (111)	1.45 (111)	3.06 (111)	1.53 (111)	1.32 (102)
3hsa-D	2.14 (112)	2.36 (112)	2.62 (112)	2.02 (112)	1.63 (103)
3hsa-E	2.14 (110)	2.49 (110)	2.39 (110)	2.00 (110)	1.20 (101)

[a]Number of aligned C^{α} atoms in parenthesis.

Table S7. Structural comparisons between PH*b*2-PH domain and monomers of PH*b*1(*sa*)/PH*b*1(*sl*)

RMSD	3dcx-A	3dcx-B	3dcx-C	3dcx-D	3dcx-E
3b77-A	3.0 (111)[a]	3.35 (105)	3.08 (108)	3.10 (107)	2.94 (97)
	3hsa-A	3hsa-B	3hsa-C	3hsa-D	3hsa-E
3b77-A	3.12 (110)	3.38 (108)	3.27 (107)	3.00 (106)	2.75 (102)

[a]Number of aligned C^{α} atoms in parenthesis.

Fig. S1.

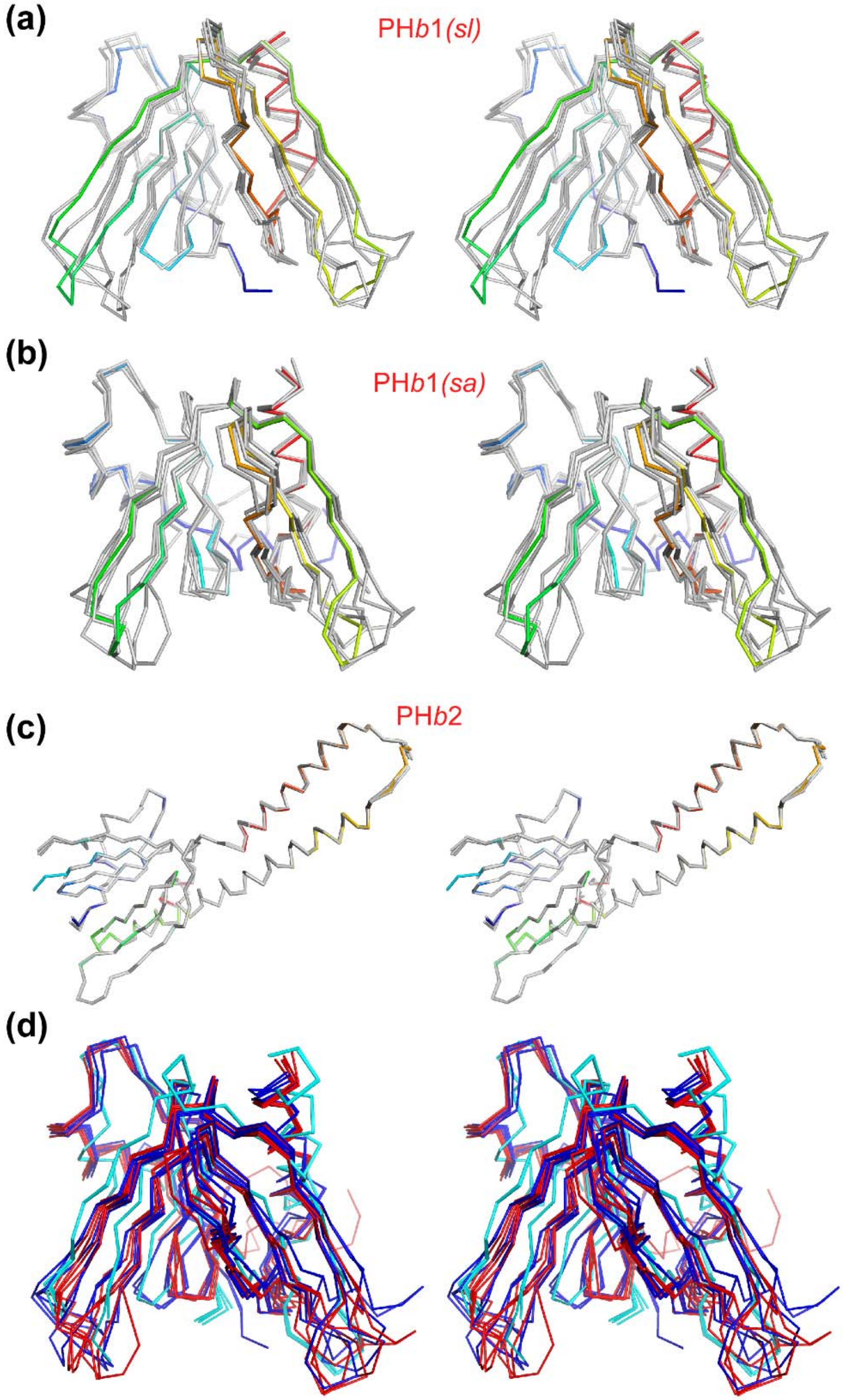

Fig. S2.

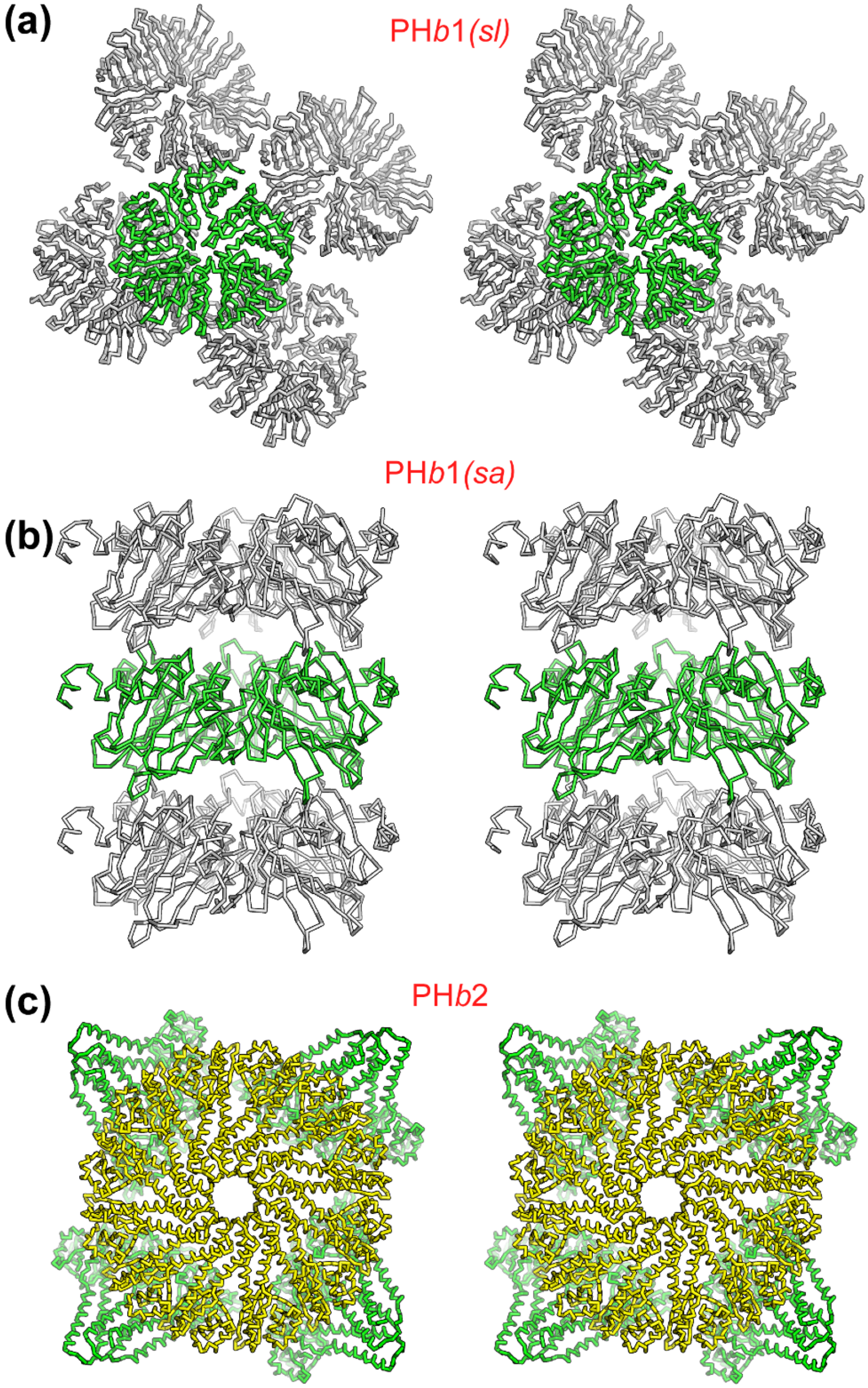

Fig. S3.

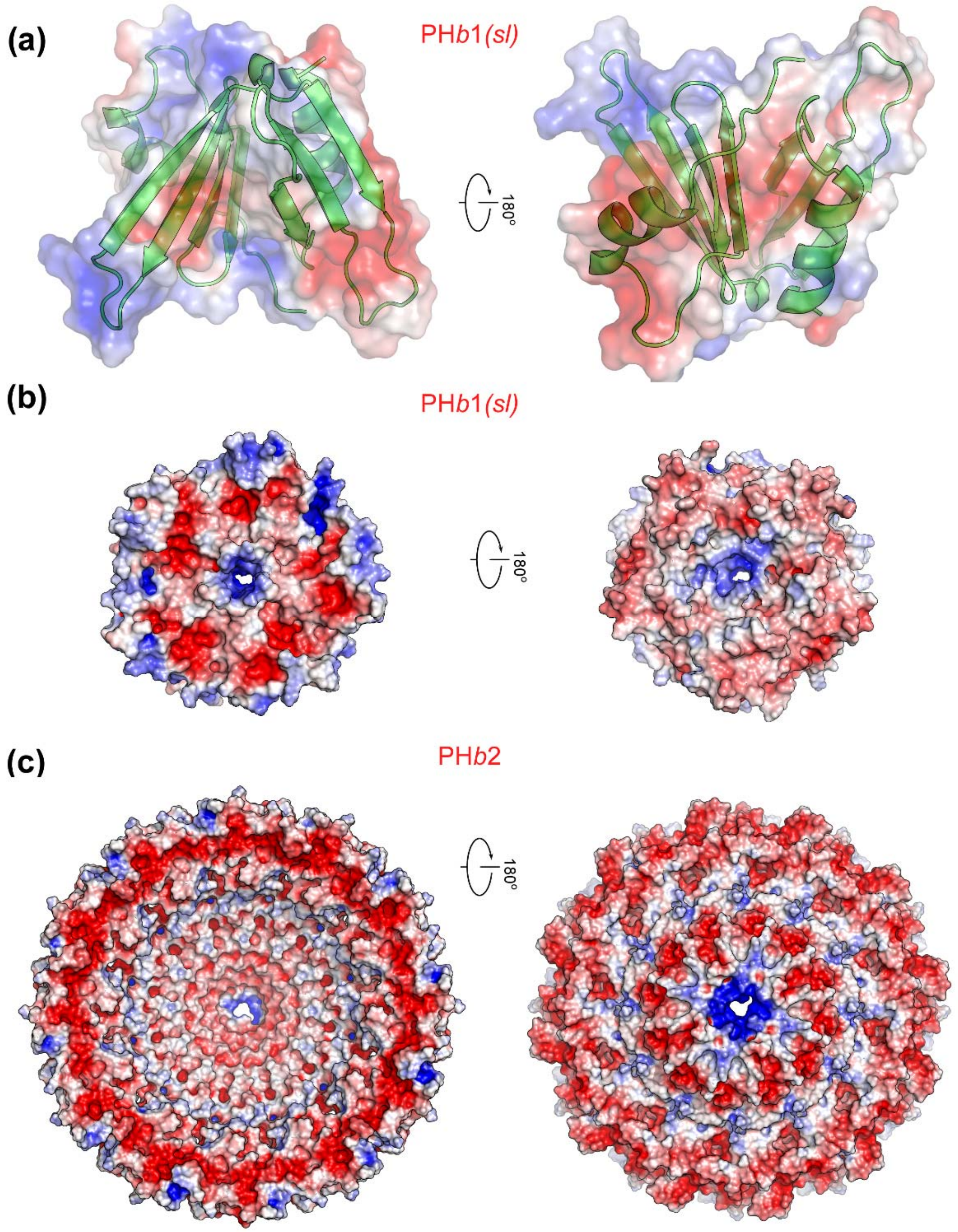

Fig. S4

(a)

```
Secondary Struct.  --EEEEEEEEEE----EEEEEEEE-EEEEEEEEE---EEEEEEE--HHHH---EEEEEEEEE--------EEEEE------HHHH-HHHH-H--
A0NKK3_1_75-160    ALFRYIFFAYRFDEN-SLTIDSGV-FVRHHEHIPFNKIQTIQHKQWFFLQPFGLESLSIETAGHND-GKAEAILPV---IPLKIS-NFIE-KLN
Q5HEC8_1_63-143    QIVGIMNTRYWIEDN-YFILTTGI-FNKKRKELNIKRIQSVDMTQGVVNQIIGGVDLQIKTP------SDGIVLSV---ISKKQG-EYLE-RYI
A0M124_1_402-482   QLLYYRSLRLSFSEE-FILKNSGV-WEKKQQYLEIWKLQAVSMSQPLWYRKKNLVNLIFHSA------GGDVRFEL---IDRNKA-ESLM-DYV
A3CWW6_1_68-152    IPLYHESIVYRLTVT-EVTWRRGV-WFRQTGIVPYNRITNVDIVQGPLMRFFSFSAVRVQTAGYS--AQAQAEIVL---NGIADP-KDLQ-ETI
A5N1P5_1_58-140    QFLAWRKNFFIVKTN-SIYHEEGI-FSIKKIEIPLDRINTIDISQKLLERIFKVATIKIDTGDTN--KDSELKFT----LDKDKT-ETLK--NI
Q721T2_1_399-484   GVASYRATGV-FTDKHTLLVQSRPIFSKLTHIIRKERIQGLSLRQSIWMEKGGSRHLNVWLKSGS--TSAEAYVRY---INQDLA-IKVY-NWY
A6CG36_1_169-250   WWLQTRFRSVTVTSK-RTLYTQGI-FSKQTSEVQHDDVRNMQVNQSFFDRLVGVGHIAISSS-----GQDDMEIDV---RGLKSP-QRVI-DII
Q2S4T2_1_276-363   HVARYYNFRLWLDGD-KLRKRHGL-FTVTEGTIPLDKVQALILRTNPFMRAFGWYELKVQTIGL(4)QGHRVIAPF---AGAERI-LELA-RQV
Q8XM54_1_452-535   SILKGRNINLKIEEN-KVHAVTGG-FFRTIHILKGKDIQAVGFNTNPIQEKNNIGKIVIDYYSE---NSEEIKLPY---MNKNYV-EVLL-NSS
Q5KZA4_1_390-472   GIRRFQIAGWSLSSK-QLALRSGW-FRQTTTYILKRHVQSLETSATWWQKRKRLATISIAVMP----LGTRVRVVD---VDEADA-ASVY-RWL
A0Y0J7_1_79-160    ASIKLQGVA--MRQH-DIAFKKGI-IWRRVTILPLARVQHIEIHRGPIERKLGLASLKLYSAGG---MSADLQVSG---LTHTDC-KNMR-QFV
Q73CA2_1_384-464   AYARYTSSGYMIRDNQLVMVYRG--LAKYTGIMRRRHVQAVGYSQSYFQKKDELCTAVVSVA------GHPYKVKH---MQKEDA-LCIY-NWY
A9NAV6_1_80-166    AYVTYRTSEYGVTDK-RVIMKKGW-IRRNSLEIFLRRLEGVDVNQTVTGRILGYGTLVISGMGGSRDYYTNVPQPL---MFRKYV-QRQV-DLL
A4ILF0_1_378-461   RIREYRHTAFRLEDT-HVQLRSGA-FTVETLVTKRAKLLELQFERSLLQRMFGVMSVKLTNR-----AHPVHVTTL---LDIDSSLQPLITSWF
Q0TSV7_1_307-394   YFIKYYNFTLLKEGE-NIKIKYGF-FSTKEFSFKENSIKLIKLKSNPLRQLLKRYEINVVIKGY(4)KEQIIMYPI---GNDKEV-QNII-REF
A0JUF8_1_82-161    RVLRWHATQYVLTSR-RIIARYGM-LRRRDIQIPLAHVHHIGVSQSLWQRILRSGNISLDTG-----QGADAVI-----PDVPEA-VRFR-NFV
A7GLR9_1_265-351   TFFTYKNFKVQRSTF-GIDISYGL-IDKKEVSIAKHDIRAVEIVQPWIYRLFGYSKIHLQLI-G(4)KSKIVLIPS---IQSSEV-SSLF-KKY
A4C2S8_1_256-345   VLLRHFNLRVYLKDN-ALEIYQGL-ITKKSIVLKKDKVQHITISHNPIKKKLGISFITFKQAVS(4)KKQDKIIKI---VGCKKEQIAIITNLL
A7B794_1_64-144    PYFRYNRYRYSINEE-CIDIREGY-LFVKRNIVPIERLHKLEISKGPIDQIFHVAKVTVTTA------GGDVTLRF---LEEEKA-EKIA-ENL
A6G2U2_1_82-163    TYVTHVSTKYKVTGR-RIETEHGV-ISKSVDSLELWRVLDVKYNQSLLDRVLGNGKITLIST-----DQSDPNLEL---HGLPNH-RELF-EKL
A7B9U4_1_402-484   DPLVYKRRAFGLTDA-VFAIRDGF-FNLRFSIIPLTRIQSVSLHQGPIQRWRRVASVRAALVP----GPVASMAEH---VEVGRS-LELW-AQL
B4RX55_1_450-530   LTLRWWRWGISYDNK-YVYIRSGR-IGIDYQCFEPHKVQQVIVKQSVFMKRRKLATIKFVLA------SGAVTVPF---LPEQYV-FTLA-NNV
A0QQT0_1_64-149    GLARWFTTTYRIEPN-EVQLRTGV-LQRKVLAVPRNRIRSVSTDARLLHRVMGLTVLRISTGQEAK-GDAEFALDA---VEAGQV-PRLR-AIL
Q8L2A0_1_48-129    RYLVIHSIRYEITTE-RIRFHRGV-LNRKMDETELYRVRDYSIRRPFHLLIFHLATLHIDTK-----DVRHSTIDM---IGIRNA-EQVL-TML
O26822_1_86-166    DVISWRNRRYIITDQ-RVMVEEGV-LWKRRYYINHRKIVDVSFSQSITERLLDSADLEIHGG------HEGTNIIL---RDAPSP-SKIE-YHI
Q3BNL4_1_405-490   AQRQVQRMAYAVDAR-YVAVRGGW-WKRWWRLAELDKLQALQLQRSPLDRLSGTATLWLDTVGASA-TGPALRLRF---VPLAQA-QALQ-AQL
A7A966_1_18-101    CRTPFTFTVYALTDK-ELSVKTGI-LNENFNLIKLFRIVDISVERTFLQRIFGMSTIVLDTRDQ---SSGNGVVAL---KNVLNG-FEVR-KVL
A7GLR9_1_62-145    SFLIWKNNVFIFYED-ILSVREGV-WSKEYSDIHYTKVKSISIDRTFVKRILNVSNVNIETVGG---DTIQIVVSN---KKLSYI-KSIL-NRH
A6PLN6_1_81-162    IILLRKNQFYTISTQ-SITSEGGV-LIRFNHTLRRNQIQSVSYTQTLIQQLLGCGNIVVSTA-----ASSRGGIIL---TNIDHV-QEIY-KAI
Q26E06_1_268-354   SINKFYDFKMELRDE-HLEVRMGL-LNKKEIKIPLSKIQILEFHSNPLRKILDFKTARIYQA-Q(4)QISSVEVPA---CHAHIQ-AQLQ-YLI
Q1VZX2_1_419-499   SILKVKKSSIGINPT-FINVRNGS-IETIHKLIEIHKLQSVKLRRNIFQQYNGHADLILETA------SGSLNIEY---LKVEEA-RKIL-NYL
Q0RP62_1_82-163    KVADWQFDHLMITDK-RLLKVSGI-FFRKVQTMPLSKITDLTYNRDPLGRLLGYGEFVVESA-----GQDQALSKI---QFLPRP-DRLY-LTL
B1YH17_1_231-317   FVLRYGSFRATRDKQ-RMTIGYGL-LNRTEIVFHQDKVQALVIEESWIKRRLKRAHLSLHII---SASGEEEKLLLHPFIRTSEI-DDFL-SRF
```

(b)

```
Secondary struct.-EEEE--E--EEEEEE-----EE E-----EEEEE---------HHHH----EEEEE--------EEEEE---HHHHHHHHHHHH-
P37523_2_60-136   LKYVL--KEHHLIIKAG-LIKHQ-IPYENIDKVVQKKKLWSG-FRLIGSRHAITIYYQ---GGWGH-AVISPQKSEEFIHKLKEKN
A6T2Q0_1_61-134   TRYTL--EASRLLVQSG-PFKWT-IPLADIKNITPSNNPLS---SPALSLDRLRIEYG----NRNA-LMISPKDKEQFLLDIEAAR
Q5WGJ5_1_65-143   IKFVL--TEEHLLIKGG-PFKRK-ILYPNIIKVVPTTDRFTG-YQISSSDKGIELRYKT-ANATRT-IKILPKDKLKFISELRKRC
Q5WLL6_1_65-138   TGYTL--SGSRLKIVYG-PIRKT-IDIHDIRTIRSKIDPFI---DPALSMDKIEINYG----QFET-ISISPKRKDEFISKLLEKN
Q6HLV3_1_52-125   TKYIV--GEETITIRSG-FVKKH-IFIRDIKQISNTKNPIA---AYALSFDRLEIVYG----AHQT-EIISPKDKEQFINHVKNKN
Q8A2X0_1_53-129   TVYTV-STDGVLEISTGRFMRKKVIPIAEITAIRKYHSMKF---GKFSVTNYVLIEYG-----NGKFASVMPVKEREFVELIKKRM
Q9K9B1_1_42-116   TYDRI--DGDRLFAISG-PFRWS-IPIQDIRSIEKRKNLLS---GPSLSLDRLTILYG---VGYDI-IVISPEKEDVFLQLLLDKN
A6KZD8_1_54-130   STYTL-TADGKLVVYYGRFYKGKTIPLTDITDVELKRSSGF---GGIMPSKYVLIHYE-----KKNLLSLVPVKPEEFINALVKRL
A5WI48_1_64-136   IKYTL--TADTLLVKNG-FSTQS-ISLEDITHITPTSSTLS---AAALSLDRIEIRYE----GGSI--VISPKDKDRFYHAIQERV
Q8ELH5_1_56-129   TRYRI--DNNTLRISYG-PMKWS-ININEIKSIRKTTNLFV---GPCLSVHRLEIHYG----NYKV-IQISPKRMQLFIKEIQKIN
A4BZG7_1_58-133   TSYKI--ENNEFIYRSG-FLRGK-IGIPNIKEILKEKTMWSG-IKPALARNGLIIKFN----KYDE-IYIAPENNNELISDLLKVN
Q9KEX6_1_65-138   TGYTV--TEDWLVIEYG-PFKKK-VEIETIESIRETKNPFI---DPALSMNKLQLYYG----NSRH-IAISPQEKEHFKKQLVKRN
Q67SW8_1_62-135   TGYAV--TENELVIRSA-FLTWR-IPLAAIRRVRPTRSPLT---SPALSMDRLEVRTN----KGSA-PLISPRNRSEFLALLRERC
A7UYW5_1_53-129   TTYTV-TPDGKLVLSFGRFSRSKEILMKDITSVERASSMQV---GRFAVMRYVLVKYG-----EGKCVVLLPVKEEEFIRLLEERR
Q813R4_1_53-128   SHYNI--TESSLVVKHGFIFHTE-IPFEDIRHVKYSGKKLH---SKKWTRQQLEIHYN----LFDSVTTFVPLEEEKFISLLKENC
Q5WM07_1_64-139   TKYVLKPDSRTIVVVFG-FYKKT-ININTINAMRCTKDFFA---SPALSSNKIEIEYS----HSNL-IRISPKEKQLFIEQVKKIN
Q8ETD0_1_64-141   LCYIF--FYDYLLVKSG-FFRFH-IKYSQMTKIEATSNFLIG-TRAMMATNGIVIYYS---SGITGELKLSPDDQDAFLKILQERA
A7AH02_1_54-129   TWYKI-TADGHLIAHCSIFPEKK-IPISEISAVEVTVMPVS---SYALSLDRLIIYKG-----DTQWLLISPVNKQDFVKLLKKHN
A4ASP1_1_60-135   TNYEL--HKDGFIYRSG-PINGK-ISIDRIYEIVKGRTLWMG-SRPATAKKGLIIKYD----AYNE-IYISPKTNEKFIEKILELN
A6KZD7_1_56-123   TIYIV-KDNGVLEIKPG--WGNR-ICVDGIRKVSYN--------PNAIGMQKVKIEHA------QGFVMINPDKPLEFVEALREID
A4CQ29_1_54-128   IRYEL--REETLHVHGSFLVKLH-IPVREIRSIRRSYNPLA---SPAGSLKRLAVRYG----DSGM-TLISPKDEADFISTLKALN
A6EQ87_1_59-133   TRYKI--LGTELQITCFPFYDKK-VAIDSIKKVAFSRSIMS---SPAPSLDRIEIFFN----TYDS-VIISPKDKEQFMDHLKQIN
A4ANC2_1_53-127   TYYVI--DGKTLKVKSGFIVNKS-FEINRIIKISETNNPIS---APAASLDRLEIKLD----SKRS-VIISPKLKHEFIEELKKIN
A3J6N6_1_59-136   TFYKI--ENTCLHWKSG-PFYGE-IDIQKINKIEYHKGIIV(4)KPALSHIGIIITYN----KYDD-IYISPEKQEEFIATLQRLN
Q64WH9_1_55-123   CKYIV-KQNGDLQIVND-FFRQK-RTFSHITDVTYT--------RHALGMQKIKIRHA------TGFVMIDPQSPRELIKALQKTN
Q5WDR5_1_61-138   ITYEL--KEEGLFVQAG-LISRF-YSYESMTALEPMGSPFSGKERIVGSSQGFNIKHN----GPKGEVKVSPERMEEFKQELLKRA
Q8EQJ6_1_52-125   TGYRI--DNDKILIYYG-PVKQT-VKIKDIEVIFKTKFPLT---SPALSFDRMQIKSG----KYDI-VTISPEEKESFLQQLMDIN
A6CHZ7_1_57-130   TCYIF--KEQKLLIRYG-PFRWR-ILIKDIKSIRKVKSPFT---SPALAVNRLEIYYQ----KYEV-VQISPQDQDAFINYLCNQN
Q7USX7_1_65-137   CRYTL--LDDALSIRCG-LICYQ-VAYADITEAIPSSTWIS---GPAMSLKRVIVRTA----KRDH--ILSPEERERFIEELMDRV
A6CKW3_1_69-152   TYYEI--NDRVLRIVAG-PIRYT-IEIERIKSVRPSRNPLS---SPALSLDRLEITYS(10)SWNT-ILISPKNKERFIDELLKVN
Q8CV26_1_61-135   TGYQI--VNDIVKVKAG-PFKKT-INIQEINKISKRKSVWT---AAALATDRLVIQYG----KYNLDILVSPRNESDFIKLLLSKK
```

(c)

```
Secondary Struct.  ----EEEEEEE----EEEEEE-----EEEEE----EEEEEEE--------EEEEEE---EEEEE------HHHHHHHHHHHH--
A3SQM8_1_77-159    RSGETREELCIDPERAVLIRSNPDGREQRWQANSYWVEARIYPKGGP-VPQYITLRGEGREVELGAFLSEPERARLITELTTAL
Q2NYF1_1_75-150    RSGEREEAIRVGEAVVEVFPSG--HAPPAFQAHPHWVRLCMERDD------RVLLVSSGKQIEIGSFLGPAERVELAMTLKRLL
Q2K411_1_76-158    RAGRVREQVTVSRTDVSVRKFAPSGRMVEHHFNPFWARFLVRRHQEI-GILSMHIFGEGRRTDIGSFLNPDDRESFAKAFRGAL
Q2J311_1_78-160    ARGRATEEISMTPSELRVRRTSPRGQVAEWVLNPLWVRLEKIVHAEF-GIEQLYLVSSGRRVSVASFLGAEEKASFANALTAAL
A4AAP7_1_72-154    WKLEYRHVITLDD-SVVSIDKGHYAPKRRWRFKRDQVALAITPEKHPWEGPGLSVHGNEETVRVGEFLNRDDCLSLMALLRKEL
A5XSB1_1_70-148    RHAVDYERIRLFP-HRLVIERMSAERLTQIELNPRWVRVEPGASPRD----PITLVSRGESVVVGQHLAQYRRAQFARELRASL
A4GJG9_1_73-153    KWSSKREKIFISQ-DKVTIEKGIHKADYRWEEFRTFTSFHVTKDINK--VLKLSFRSKGEDVEVGAFLNEDDKNVLKEEVSNII
A7IFT4_1_85-167    RSARAREHILVTPSVIEVRREPARGRRTITRLNPFWTRLTREDDEDH-GTLDVALVSGPRTVPVGRFLGPDQKAALATDLSRAL
Q168L9_1_76-158    KARNIVEVLTLNDEEARLIRTEPTGATREWDCNRYWTTITKYEKDGP-IPHYVTLKGMGREVEIGAFLSEEERVALYDELQRAW
Q60AH5_1_74-156    RQSAVREVITVTD-ADVMLERGIRGPDETYRFRRAWLGVSLDGPAAAGHPSRLCLKRHGRKIEIGRFLVESEREALYRELKKEL
Q0C0K4_1_71-152    RQQQEETRVTVTARAICLHHKDAKGREKRAELPSAFARVELEEPAGP--ASWLRIEHGKTAWIIGRFLTPPERSDFAKALRQAL
Q7W345_1_81-163    RHARDGEDIELRADGTLVVEVHDGERVSRHVFDRGRARVIRHRAWSA-ADESLWLHCGRRQVRLARYLDRRRTCAFETDIRQAL
A3RSA3_1_84-162    LHTSDHERIELDD-DALVIEQVFANQRVRHVFNPRWVRVELGEPLRE----QVALCSSGRVVRVGRFLDPAGRRRLADELSRCL
A3UIN0_1_88-169    RDGRRMESIKITREEIRVIRRFPTGHLIQFVLPSAWTRVIVEGEGEP--DVQTRLTAMGKSLIVGSWLSPREREESLADAIRDAL
A1VTB4_1_93-172    LHAADGEQISFSPEGQLAIEVVRGLDTRHYRMNPAWAHLERGGPRKD----RLWLCCSPLRVEVATQLGAGEKRRVERELKQAL
Q9A236_1_68-149    RAARRVERIQVTAEAVTVSREDEKGARTVWTSPTAFTRVGVEQPGEH--EVRVRLMIHRKRLTLARALGPDQRLEFGAALQDAI
A2SKP6_1_95-173    RHAADRELITLLP-GRLVVEHLNGGRIERAEFVPDWVRVEPRDDDRS----LIELSGQGRVIAVGRYVRPELRRALAEEFRTAL
Q0BPC5_1_89-170    RAVRQTEIVTLSGTGPGIAHIDARGRSRHMKIRPGWLRLRLEECPGR--VPQLILSSRDGEFELARSLGEDEKRALAETLQDAL
A4BQS4_1_74-156    QRAYDTEVVHVSE-SKVEIDKGRRRPERHWSFDRLWSEVILAGPGHPWYPTRLAVRSRGEQVELGRFLADEERARVAGELRRWI
A4SZC5_1_63-141    RHALDCETIEIDG-TRLIVKKFIGYKETIYEFNSRWAKIEPPIAGSK----TFHIIQSNLRVELGQFIRHEQMALIASIRPHL
A0P3P2_1_83-165    HSARTFEEVVVSRHEIAIRKVGPGKKYQEYRFNPFWVRLTVDRIEDE-GVVKVTLQSRGEKVDLGNFLNPDDRTSFAGAMANAL
A3NFM1_1_145-225   RHAVDYDCVALTE-QRLEVIQCDGAQLRRYDWNPLWVAVDLDAAHAR--DPTIRIRHGSETALVGRHVTLARRRHVARELNAAL
A1B3D8_1_80-159    GTAR--EVMLLDRDRLILTRSDPGRPDRIWQTNPYWVRLALRQN-GP-VEDYLVLTDGKREVELGAFLAPEERMALRNDLARRL
A5G1G0_1_85-166    RGAKASEVIVLTDEALTITRTTPGGRRSEVRLEAGWLRVDVEEQAGT-N-PIVSVANREARQIVGMALGDAERRDFADALKAAI
A3VQB4_1_95-176    AQGRRHERLILTDDALWVIRVLPSGHETRWKLTPAFVRIDIARPIEH--DSQLCLRECGKTLVIGSFLAPKERGEVAEALERVL
A3VHI7_1_90-172    RDGEILEELTIWTDRMHLSRTGPRRQHAEWDANPHWVSVQVHKDGGP-VKHYLTLKGNGREVEIGSFLSEDERPLLREELERAL
A6F2F8_1_72-154    RRCQRREVLTFAP-ELIRLEKGLTRKEQEWELPRRHTRVWQDMPRHPWTPPKLHLQFRGEEISLAPFLNIDDTEELVAILERHG
Q2YZX7_1_124-204   RRTDVIETVEISP-RDITVHRRELGREETKVFPAYWAHVDFSGSPTQ--NGTLEIRSHGEAIEIGRFLSASEKDRTAWKLNDVL
A6GLS2_1_97-175    RHAADYEMIELQP-NQLTLVMADGTKLTQLEWSPQWAKLSYNGKYKA----PLLFSHKGQQVKIGKFIAEKDKSALHRELKAAL
A7HZ34_1_77-158    KAARAHETVQLTDDELLVRRVDAKGRARAFAFQPYWVRLALRKEPDE--TTHLHLLSHGRQLEVAAALSPPERESFMHALEAAL
Q3J6Q7_1_74-156    RRAQHCEIITIGQ-EEIEIFRGRETTGETWKFHRYWARVRIELPPYAWHMSRLIIGSHGHEVEIGVFLSEEERLRLAKELQAVC
B7L1X4_1_84-166    RRGRSFEEVAISPLEVFLARIDPRGVRREWRFNPLWTKLSRIDDDEF-GLRTLTLTSRREHVVVARDASPDERAIVADGLTRAL
Q28SZ7_1_86-168    RDMDLYEDVMIWDDLIRVERHERRHALRDWEANPYWVRMVLHAKGGP-VPNYLTLQGGPREVELGAFLTPLERVELKQLLDRNL
A1TT19_1_80-163    RHALDGDLIVLLDNGELEICCLRGAQEQHYRFPAAWCRVECVPGRHRAERSGLCIACGRHRIALGAWGSPRRADRLAGEIRAAA
Q2SMB1_1_74-155    KRCASQEVILITP-LEVCIEKGMAQPERTWTFPRWYTRIILVEGGRN-GHICVMIACKGEEVEIGAWLAEGDRKALIATLRSLV
A3JQ23_1_89-171    LDGQLREVLHIWEDRITLTHIPRKGTAAFWQANPYWVKLVKHDTGGR-VPEYLTLEGSGKIVELGAFLAPEERRELYIYLNRAL
Q3SLX6_1_78-159    ERKDDYERLTIDG-DRVVLEWRSRKREGRRELNRQWTRVRCTCAAPG-RNCRVGVCCYGRETLVGQYLSDEARLRLAATLRSKL
```
