# Supplementary material for: Bacterial Pleckstrin Homology Domains: A Prokaryotic Origin for the PH Domain
Source: J Mol Biol. 2010 Feb 12;396(1):31–46. doi: 10.1016/j.jmb.2009.11.006 (PMC2817789; doi:10.1016/j.jmb.2009.11.006)
Supplement: Supplementary Fig. 4 [file mmc2.doc]

**Fig. S4**

(**a**)

Secondary Struct --EEEEEEEEEE----EEEEEEEE-EEEEEEEEE---EEEEEEE--HHHH---EEEEEEEEE--------EEEEE------HHHH-HHHH-H--

A0NKK3_1_75-160 A**LF**R**YI**FFA**Y**R**FD**E**N**-S**L**T**ID**S**GV**-**F**V**RH**HEH**I**P**F**N**KIQ**T**IQHKQ**WF**F**L**Q**P**F**G**L**E**SL**S**I**ETAGHND-GKAE**A**I**L**P**V**---IPLKI**S**-NF**IE**-KLN

Q5HEC8_1_63-143 Q**IV**G**IM**NTR**Y**W**IE**D**N**-YFI**LT**T**GI**-**F**N**KK**RKE**L**N**I**K**RIQ**S**VDMTQG**VVN**QII**G**GVDL**Q**I**KTP------SDG**I**V**L**S**V**---ISKKQ**G**-EY**LE**-RY**I**

A0M124_1_402-482 Q**LL**Y**YR**SLR**L**S**FS**E**E**-F**I**LK**N**S**GV**-**W**E**KK**QQY**L**E**I**W**KLQ**A**VSMSQP**L**W**Y**RKK**N**LVNL**IFHSA------GGD**V**R**F**E**L**---IDRNK**A**-E**SLM**-DY**V**

A3CWW6_1_68-152 IP**L**Y**HE**SIV**Y**R**LT**V**T**-E**V**T**WR**R**GV**-**W**F**RQ**TGI**V**P**Y**N**RIT**N**VDI**V**QG**P**L**M**RFF**S**FS**A**V**R**V**QTAGYS--AQAQ**A**E**I**V**L**---NGIAD**P**-K**DLQ**-ET**I**

A5N1P5_1_58-140 Q**FL**A**WR**KNF**F**IV**K**T**N**-S**I**Y**HE**E**GI**-**F**SI**K**KIE**I**P**L**D**RIN**T**IDISQ**KL**L**E**RIF**K**VATI**K**I**DTGDTN--KDSE**L**K**F**T----LDKDK**T**-E**TLK**--N**I**

Q721T2_1_399-484 G**VA**S**YR**ATGV-**FT**D**K**HT**L**L**VQ**SRPI**F**S**K**LTHI**I**R**K**E**RIQ**G**LSLRQS**I**W**M**EK**GGSR**HL**N**V**WLKSGS--TSAE**A**Y**V**R**Y**---INQDL**A**-I**KVY**-NW**Y**

A6CG36_1_169-250 W**WL**QT**R**FRSVTV**T**S**K**-RTL**YT**Q**GI**-**F**S**KQ**TSE**V**Q**H**DD**VR**NM**QVNQS**F**F**D**RL**VG**VGHI**A**I**SSS-----GQDD**M**E**I**D**V**---RGLKS**P**-Q**RVI**-DI**I**

Q2S4T2_1_276-363 H**VA**R**YY**NFR**L**W**LD**G**D**-K**L**RK**R**H**GL**-**F**TV**T**EGT**I**P**L**D**KVQ**A**L**I**LRTN**P**F**M**R**A**F**G**W**Y**EL**K**V**QTIGL(4)QGHR**V**I**A**P**F**---AGAERI-L**EL**A-RQ**V**

Q8XM54_1_452-535 S**IL**KG**R**NIN**L**K**IE**E**N**-K**V**H**A**VT**GG**-**F**F**RT**IHI**L**KGKD**IQ**A**V**G**FNTN**P**I**Q**EK**NN**IGKI**V**I**DYYSE---NSEE**I**K**L**P**Y**---MNKNY**V**-EV**LL**-NSS

Q5KZA4_1_390-472 G**I**RR**FQ**IAG**W**S**LS**S**K**-Q**L**A**LR**S**GW**-**F**R**QT**TTY**I**L**K**R**HVQ**S**LETS**A**T**W**W**Q**KRK**R**LATI**S**I**AVMP----LGTR**V**R**V**VD---VDEAD**A**-A**SVY**-RW**L**

A0Y0J7_1_79-160 AS**I**K**LQ**GVA--**MR**Q**H**-D**I**A**FK**K**GI**-**I**W**RR**VTI**L**P**L**A**RVQ**H**IEIHRG**P**I**E**RKL**G**LASL**K**L**YSAGG---MSAD**L**Q**V**S**G**---LTHTD**C**-K**N**M**R**-QF**V**

Q73CA2_1_384-464 A**YA**R**Y**TSSG**Y**M**IR**D**N**QL**V**M**V**YR**G**--**L**A**K**YTGI**M**R**R**R**HVQ**A**V**G**YSQS**Y**F**Q**KK**DE**LCT**AV**V**SVA------GHP**Y**K**V**K**H**---MQKED**A**-L**CIY**-NW**Y**

A9NAV6_1_80-166 A**YV**T**YR**TSE**Y**GV**T**D**K**-R**V**I**MK**K**GW**-**I**R**RN**SLE**I**F**L**R**RLE**G**VDVNQT**VTG**RIL**G**YGTL**V**I**SGMGGSRDYYTN**V**PQP**L**---MFRKY**V**-Q**R**QV-DL**L**

A4ILF0_1_378-461 R**I**RE**YR**HTA**F**R**LE**D**T**-H**V**Q**LR**S**GA**-**F**TV**E**TLV**T**K**R**A**KL**LE**LQFERS**L**L**Q**RMF**G**V**M**SV**K**L**TNR-----AHPV**H**V**T**T**L**---LDIDS**S**LQP**LI**TSW**F**

Q0TSV7_1_307-394 Y**FI**K**YY**NFT**L**L**KE**G**E**-N**I**K**IK**Y**GF**-**F**S**TK**EFS**F**K**E**NS**IK**L**IKLKSN**P**L**R**QLL**KRY**EI**N**V**VIKGY(4)KEQI**I**M**Y**P**I**---GNDKE**V**-Q**NII**-RE**F**

A0JUF8_1_82-161 R**VL**R**WH**ATQ**Y**V**LT**S**R**-R**I**I**AR**Y**GM**-**L**R**RR**DIQ**I**P**L**A**HVH**H**I**G**VSQS**L**W**Q**RIL**RS**GNI**S**L**DTG-----QGAD**A**V**I**-----PDVPE**A**-V**R**F**R**-NF**V**

A7GLR9_1_265-351 T**FF**T**YK**NFKVQ**RS**TF-G**I**D**IS**Y**GL**-**I**D**KK**EVS**I**A**K**HD**IR**A**VEI**V**QP**W**I**Y**RLF**G**YSKI**H**L**QLI-G(4)KSKI**V**L**I**PS---IQSSE**V**-S**SLF**-KK**Y**

A4C2S8_1_256-345 V**LL**R**HF**NLRVY**LK**D**N**-A**L**E**I**YQ**GL**-**I**T**KK**SIV**L**K**K**D**KVQ**H**ITISHN**P**I**K**KKL**G**IS**F**I**TFKQAVS(4)KKQDKI**I**K**I**---VGCKKEQIA**II**TNL**L**

A7B794_1_64-144 P**YF**R**Y**NRYR**Y**S**IN**E**E**-C**I**D**IR**E**GY**-**L**FV**K**RNI**V**P**I**E**RLH**K**LEISKG**P**I**D**QIF**H**VAKV**T**V**TTA------GGD**V**T**L**R**F**---LEEEK**A**-E**KI**A-EN**L**

A6G2U2_1_82-163 T**YV**T**H**VSTK**Y**KV**T**G**R**-R**I**E**TE**H**GV**-**I**S**KS**VDS**L**E**L**W**RV**LD**VKYNQS**L**L**D**R**V**L**GN**GKI**T**L**IST-----DQSDPN**L**E**L**---HGLPNH-R**ELF**-EK**L**

A7B9U4_1_402-484 DP**L**V**YK**RRA**F**G**LT**DA-VFA**IR**D**GF**-**F**NL**R**FSI**I**P**L**T**RIQ**S**VSLHQG**P**I**Q**RWR**R**VASV**RAALVP----GPVASM**A**E**H**---VEVGR**S**-L**ELW**-AQ**L**

B4RX55_1_450-530 L**TL**R**WW**RWG**I**S**YD**N**K**-Y**V**Y**IR**S**G**R-**I**GI**D**YQC**F**EPH**KVQ**Q**V**I**VKQS**V**F**M**KRR**K**LATI**KFVLA------SGA**V**T**V**P**F**---LPEQY**V**-F**TL**A-NN**V**

A0QQT0_1_64-149 G**LA**R**WF**TTT**Y**R**IE**P**N**-E**V**Q**LR**T**GV**-**L**Q**RK**VLA**V**P**R**N**RIR**S**VSTD**ARL**L**H**R**V**M**G**LT**V**L**R**I**STGQEAK-GDAE**F**A**L**D**A**---VEAGQ**V**-P**RLR**-AI**L**

Q8L2A0_1_48-129 R**YL**V**IH**SIR**Y**E**IT**T**E**-R**I**R**FH**R**GV**-**L**N**RK**MDE**T**E**L**Y**RVR**DY**SIRRP**F**H**LL**IF**H**LATL**H**I**DTK-----DVRHST**I**D**M**---IGIRN**A**-E**QVL**-TM**L**

O26822_1_86-166 D**VI**S**WR**NRR**Y**I**IT**D**Q**-R**V**M**VE**E**GV**-**L**W**KR**RYY**I**N**H**R**KI**VD**VSFSQS**ITE**RLL**DS**ADL**E**I**HGG------HEG**T**N**I**I**L**---RDAPS**P**-S**KIE**-YH**I**

Q3BNL4_1_405-490 AQRQV**Q**RMA**Y**AV**D**A**R**-Y**V**A**VR**G**GW**-**W**K**R**WWRL**A**E**L**D**KLQ**A**LQLQRS**P**L**D**RL**SG**TATL**W**L**DTVGASA-TGPA**L**R**L**R**F**---VPLAQ**A**-QA**LQ**-AQ**L**

A7A966_1_18-101 CR**T**P**F**TFTV**Y**A**LT**D**K**-E**L**S**VK**T**GI**-**L**N**EN**FNL**I**K**L**F**RI**VD**ISVERT**F**L**Q**RIF**G**MSTI**V**L**DTRDQ---SSGN**G**V**V**A**L**---KNVLN**G**-F**EVR**-KV**L**

A7GLR9_1_62-145 S**FL**I**WK**NNV**F**I**F**YE**D**-I**L**S**VR**E**GV**-**W**S**KE**YSD**I**H**Y**T**KVK**S**ISIDRT**FVK**RIL**N**VSNV**N**I**ETVGG---DTIQ**I**V**V**SN---KKLSYI-K**SIL**-NR**H**

A6PLN6_1_81-162 I**IL**L**RK**NQF**Y**T**IS**T**Q**-S**I**TS**E**G**GV**-**L**I**R**FNHT**L**R**R**NQ**IQ**S**VSYTQT**L**I**Q**QLL**G**CGNI**V**V**STA-----ASSR**G**G**I**I**L**---TNIDH**V**-Q**EIY**-KA**I**

Q26E06_1_268-354 S**I**NK**FY**DFK**M**E**LR**D**E**-H**L**E**VR**M**GL**-**L**N**KK**EIK**I**P**L**S**KIQ**I**LEFHSN**P**L**R**KIL**D**F**K**T**AR**I**YQA-Q(4)QISS**V**E**V**P**A**---CHAHIQ-A**QLQ**-YL**I**

Q1VZX2_1_419-499 S**IL**KV**K**KSS**I**G**IN**P**T**-F**I**N**VR**N**G**S-**I**E**T**IHKL**I**E**I**H**KLQ**S**VKLRRN**I**F**Q**QY**NG**HADL**I**L**ETA------SGS**L**N**I**E**Y**---LKVEE**A**-R**KIL**-NY**L**

Q0RP62_1_82-163 K**VA**D**WQ**FDH**L**M**IT**D**K**-R**L**LKVS**GI**-**F**F**RK**VQT**M**P**L**S**KIT**D**LTYNRD**P**L**G**RLL**G**YGE**FV**V**ESA-----GQDQ**A**LSK**I**---QFLPR**P**-D**RLY**-LT**L**

B1YH17_1_231-317 F**VL**R**Y**GSFRAT**RD**K**Q**-RMT**I**GY**GL**-**L**N**RT**EIV**F**H**Q**D**KVQ**A**L**V**IEES**W**I**K**RRL**KR**AHL**S**L**HII---SASGEEEK**L**L**L**HPFIRTSEI-D**D**F**L**-SR**F**

(**b**)

Secondary struct -EEEE--E--EEEEEE-----EE E-----EEEEE---------HHHH----EEEEE--------EEEEE---HHHHHHHHHHHH-

P37523_2_60-136 LK**Y**V**L**--KE**H**H**L**I**I**KA**G**-L**IK**H**Q**-**I**P**YENIDKV**VQ**K**KKL**W**SG-FRLI**GS**R**H**A**I**T**IYY**Q---GGWGH-**A**V**ISPQKSEEFIH**K**LKEKN**

A6T2Q0_1_61-134 **T**R**Y**T**L**--EA**S**R**L**L**V**QS**G**-P**FK**W**T**-**I**P**L**A**DIKNI**TP**S**N**N**P**L**S---SP**ALS**L**D**R**LRIEYG**----NR**N**A-**L**M**ISPKDKEQFL**LD**IE**AAR

Q5WGJ5_1_65-143 IKFV**L**--TE**E**H**L**L**I**KG**G**-P**FK**R**K**-**I**L**Y**P**NI**I**KV**VP**T**T**D**R**F**TG-YQI**S**S**S**D**K**G**IE**L**RY**KT-ANAT**R**T-**I**K**I**L**PKDK**L**KFIS**E**LRKRC**

Q5WLL6_1_65-138 **T**G**Y**T**L**--SG**S**R**L**K**I**VY**G**-P**IR**K**T**-**I**D**IHDIRTI**RS**K**I**D**P**F**I---DP**ALS**M**D**K**IEI**N**YG**----QF**E**T-**I**S**ISPKRKDEFIS**K**LLEKN**

Q6HLV3_1_52-125 **T**K**Y**I**V**--GE**E**TIT**I**RS**G**-F**VK**K**H**-**I**F**IRDIKQI**SN**T**K**N**P**I**A---AY**ALS**F**D**R**LEI**V**YG**----AH**Q**T-EI**ISPKDKEQFIN**H**VK**N**KN**

Q8A2X0_1_53-129 **T**V**Y**T**V**-STDGV**L**E**I**ST**G**RF**MR**K**K**V**I**P**I**A**EIT**A**I**RKYH**S**M**K**F---GKFSVT**N**Y**VLIEYG**-----NGKF**A**S**V**M**P**V**KEREFVE**L**IKKR**M

Q9K9B1_1_42-116 **T**YDR**I**--DG**D**R**L**FAIS**G**-P**FR**W**S**-**I**P**IQDIRSI**EK**R**K**N**L**L**S---GP**SLS**L**D**R**L**T**ILYG**---VGY**D**I-**I**V**ISPEKED**V**FLQ**L**LLDKN**

A6KZD8_1_54-130 **S**T**Y**T**L**-TADGK**L**V**V**YY**G**RF**YK**G**K**T**I**P**LTDITDV**EL**K**R**S**SGF---GGI**M**PS**K**Y**VLIHY**E-----K**K**NL**L**S**LVP**V**K**P**EEFIN**A**L**V**KR**L

A5WI48_1_64-136 IK**Y**T**L**--TA**D**T**L**L**V**KN**G**-FS**T**Q**S**-**I**S**LEDITHI**TP**T**S**S**T**L**S---AA**ALS**L**D**R**IEIRY**E----GG**S**I--V**ISPKDKDRF**Y**H**A**IQERV**

Q8ELH5_1_56-129 **T**R**Y**R**I**--DN**N**T**L**R**I**SY**G**-P**MK**W**S**-**I**N**INEIKSI**RK**T**T**N**L**F**V---GP**CLS**V**H**R**LEIHYG**----NY**K**V-**I**Q**ISPKR**M**Q**L**FIK**E**IQKIN**

A4BZG7_1_58-133 **T**S**Y**K**I**--EN**N**EFIYRS**G**-F**LR**G**K**-**I**G**I**P**NIKEI**LK**E**K**T**M**W**SG-IKP**ALA**R**N**G**LIIKFN**----KY**D**E-**I**Y**IAPENNNE**L**IS**D**LLK**V**N**

Q9KEX6_1_65-138 **T**G**Y**T**V**--TE**D**W**L**V**I**EY**G**-P**FK**K**K**-**V**E**IETIESI**RE**T**K**N**P**F**I---DP**ALS**M**N**K**LQ**L**YYG**----NS**R**H-**I**A**ISPQEKEHF**K**K**Q**L**V**KRN**

Q67SW8_1_62-135 **T**G**Y**A**V**--TE**N**E**L**V**I**RSA-F**LT**W**R**-**I**P**L**AA**IRRV**RP**T**R**S**P**L**T---SP**ALS**M**D**R**LE**V**R**T**N**----KG**S**A-PL**ISPRNRSEFL**AL**LRERC**

A7UYW5_1_53-129 **T**T**Y**T**V**-TPDGK**L**V**L**SF**G**RFS**R**S**K**E**I**L**MKDITSV**ERAS**S**M**Q**V---GRF**A**VM**R**Y**VL**V**KYG**-----EGKC**V**V**L**L**P**V**KEEEFIR**L**LEER**R

Q813R4_1_53-128 **S**H**Y**N**I**--TE**S**S**L**V**V**KH**G**FI**FH**T**E**-**I**P**FEDIRHV**KY**S**GKK**L**H---SKK**W**TR**Q**Q**LEIHYN**----LF**D**SV**T**TF**VP**L**EEEKFIS**L**LKE**N**C**

Q5WM07_1_64-139 **T**K**Y**V**L**KPDS**R**TIV**V**VF**G**-F**YK**K**T**-**I**N**INTIN**AMRC**T**K**D**F**F**A---SP**ALS**S**N**K**IEIEYS**----HS**N**L-**I**R**ISPKEKQ**L**FIE**Q**VKKIN**

Q8ETD0_1_64-141 LC**Y**IF--FY**D**Y**L**L**V**KS**G**-F**FR**F**H**-**I**K**YSQ**M**TKI**EA**T**S**N**F**L**IG-TRAM**MA**T**N**G**I**V**IYYS**---SGI**T**GE**L**K**LSPDDQD**A**FLK**I**LQERA**

A7AH02_1_54-129 **T**W**Y**K**I**-TADGH**L**IAHCSIFP**E**K**K**-**I**P**ISEIS**A**V**EV**T**VMPVS---SY**ALS**L**D**R**LIIY**K**G**-----D**T**QW**L**L**ISP**V**NKQDFVK**L**LKKHN**

A4ASP1_1_60-135 **T**N**Y**E**L**--HK**D**GFIYRS**G**-P**IN**G**K**-**I**S**IDRI**Y**EI**VKGR**T**L**W**MG-SRP**ATA**K**K**G**LIIKYD**----AY**N**E-**I**Y**ISPKTNEKFIE**K**ILELN**

A6KZD7_1_56-123 **T**I**Y**I**V**-KDNGV**L**E**I**KP**G**--**W**GN**R**-**I**CV**D**G**IRKV**SY**N**--------PN**AIG**M**Q**K**VKIEHA**------**Q**GF**V**M**INPDK**PL**EFVE**A**LREID**

A4CQ29_1_54-128 IR**Y**E**L**--RE**E**T**L**H**V**HGSFL**VK**L**H**-**I**PV**REIRSI**RR**S**Y**N**P**L**A---SP**AGS**L**K**R**L**AV**RYG**----DSGM-**T**L**ISPKDE**A**DFIS**T**LK**A**LN**

A6EQ87_1_59-133 **T**R**Y**K**I**--LG**T**E**L**Q**I**TCFPF**YD**K**K**-**V**A**IDSIKKV**AF**S**R**S**I**M**S---SP**A**P**S**L**D**R**IEIFFN**----TY**D**S-**V**I**ISPKDKEQF**M**D**H**LK**Q**IN**

A4ANC2_1_53-127 **T**Y**Y**V**I**--DG**K**T**L**K**V**KS**G**FI**VN**K**S**-FE**INRI**I**KI**SE**T**N**N**P**I**S---AP**AAS**L**D**R**LEIK**L**D**----SK**R**S-**V**I**ISPK**L**KHEFIE**E**LKKIN**

A3J6N6_1_59-136 **T**F**Y**K**I**--EN**T**C**L**HWKS**G**-P**F**YG**E**-**I**D**IQKINKI**EY**H**K**G**I**I**V(4)KP**ALS**HIG**III**T**YN**----KY**D**D-**I**Y**ISPEKQEEFI**AT**LQRLN**

Q64WH9_1_55-123 **C**K**Y**I**V**-KQNGD**L**Q**I**VND-F**FR**Q**K**-RT**FSHITDV**TY**T**--------RH**ALG**M**Q**K**IKIRHA**------**T**GF**V**M**IDPQS**P**RE**L**IK**A**LQK**T**N**

Q5WDR5_1_61-138 IT**Y**E**L**--KE**E**G**L**F**V**QA**G**-L**IS**RF-YS**YES**M**T**A**L**EPMG**S**P**F**SGKERI**VGS**S**Q**GFN**IKHN**----GP**K**GE**V**K**VSPER**M**EEF**K**Q**E**LLKRA**

Q8EQJ6_1_52-125 **T**G**Y**R**I**--DN**D**KIL**I**YY**G**-P**VK**Q**T**-**V**K**IKDIE**V**I**FK**T**KFP**L**T---SP**ALS**F**D**RM**QIK**S**G**----KY**D**I-**V**T**ISPEEKESFLQ**Q**LMDIN**

A6CHZ7_1_57-130 **T**C**Y**IF--KE**Q**K**L**L**I**RY**G**-P**FR**W**R**-**I**L**IKDIKSI**RKVK**S**P**F**T---SP**ALA**V**N**R**LEIYY**Q----KY**E**V-**V**Q**ISPQDQD**A**FIN**Y**L**CN**QN**

Q7USX7_1_65-137 **C**R**Y**T**L**--LD**D**A**L**S**I**RC**G**-L**IC**Y**Q**-**V**A**Y**A**DITE**AIP**S**S**T**W**I**S---GP**AMS**L**K**R**VI**V**R**T**A**----KR**D**H--I**LSPEERERFIE**E**LMDRV**

A6CKW3_1_69-152 **T**Y**Y**E**I**--ND**R**V**L**R**I**VA**G**-P**IR**Y**T**-**I**E**IERIKSV**RP**S**R**N**P**L**S---SP**ALS**L**D**R**LEI**T**YS**(10)SW**N**T-**I**L**ISPKNKERFID**E**LLK**V**N**

Q8CV26_1_61-135 **T**G**Y**Q**I**--VN**D**IVK**V**KA**G**-P**FK**K**T**-**I**N**IQEINKI**SK**R**K**S**V**W**T---AA**ALA**T**D**R**L**V**IQYG**----KY**N**LD**I**L**VSPRNESDFIK**L**LL**S**K**K

(**c**)

Secondary Struct ----EEEEEEE----EEEEEE-----EEEEE----EEEEEEE--------EEEEEE---EEEEE------HHHHHHHHHHHH--

A3SQM8_1_77-159 **RSG**ETR**E**E**L**C**I**DPERA**VL**I**R**SNPDGREQ**RWQA**NSY**WVE**ARIYPKGGP-VPQY**I**T**L**RGEG**REV**E**LG**A**FLS**EP**ER**AR**LI**TE**LT**T**AL**

Q2NYF1_1_75-150 **RSG**ERE**E**A**I**R**V**GEAVVE**V**FPSG--HAPPA**FQA**HPH**WVRL**CMERDD------R**V**L**L**VSSG**KQI**E**IG**S**FLG**PA**ER**VE**LA**MT**LK**R**LL**

Q2K411_1_76-158 **R**A**G**RVR**E**Q**V**T**V**SRTDVS**V**R**K**FAPSGRMV**EHHF**NPF**WAR**FLVRRHQEI-GILSMH**I**FGEG**RR**TD**IG**S**FLN**PD**DR**ES**FA**KAF**R**G**AL**

Q2J311_1_78-160 A**RG**RAT**E**E**I**SMTPSELR**V**R**R**TSPRGQVA**EW**V**L**NPL**WVRL**EKIVHAEF-GIEQ**L**Y**L**VSSG**RRV**S**VA**S**FLG**AE**EK**AS**FA**NA**LT**A**AL**

A4AAP7_1_72-154 W**K**LEYRHV**I**T**L**DD-SV**V**SI**D**KGHYAPKR**RWRF**KRDQ**V**A**L**AITPEKHPWEGPG**L**S**V**HGNE**ETV**R**VG**E**FLN**RD**D**CLS**LM**AL**LR**KE**L**

A5XSB1_1_70-148 **RHA**VDY**E**R**I**R**L**FP-HR**LV**I**E**RMSAERLT**QIEL**NPR**WVRV**EPGASPRD----P**I**T**L**VSRG**ESV**V**VG**Q**HLA**QY**RR**AQ**FA**RE**LR**AS**L**

A4GJG9_1_73-153 **K**W**S**SKR**E**K**I**F**I**SQ-DK**VT**I**E**KGIHKADY**RWE**EFRT**FT**SFHVTKDINK--VLK**L**SFRSKG**EDV**E**VG**A**FLN**ED**DK**NV**L**KEE**VS**N**II**

A7IFT4_1_85-167 **RSA**RAR**E**H**I**L**V**TPSVIE**V**R**R**EPARGRRTIT**RL**NPF**WTRL**TREDDEDH-GTLD**V**A**L**VSGP**RTV**P**VG**R**FLG**PDQ**K**AA**LA**TD**LS**R**AL**

Q168L9_1_76-158 **K**ARNIV**E**V**L**T**L**NDEEAR**L**I**R**TEPTGATR**EWDC**NRY**WT**T**I**TKYEKDGP-IPHY**V**T**L**KGMG**REV**E**IG**A**FLS**EE**ER**VA**LY**DE**LQ**R**A**W

Q60AH5_1_74-156 **RQS**AVR**E**V**I**T**V**TD-AD**VM**L**E**RGIRGPDE**TYRF**RRA**W**LG**V**SLDGPAAAGHPSR**L**C**L**KRHG**RKI**E**IG**R**FLV**ES**ER**EA**LY**RE**LK**KE**L**

Q0C0K4_1_71-152 **RQ**QQEETR**V**T**V**TARAI**CL**H**H**KDAKGREK**R**A**EL**PSA**FARV**ELEEPAGP--ASW**L**R**I**EHGKTAWI**IG**R**FLT**PP**ER**SD**FA**KA**LR**Q**AL**

Q7W345_1_81-163 **RHA**RDG**E**D**I**E**L**RADGT**LV**V**E**VHDGERVS**RH**V**F**DRGR**ARV**IRHRAWSA-ADES**L**W**L**HCGR**RQV**R**LA**R**YLD**RR**R**TCA**F**ETD**IR**Q**AL**

A3RSA3_1_84-162 L**HT**SDH**E**R**I**E**L**DD-DA**LV**I**E**QVFANQRV**RH**V**F**NPR**WVRV**ELGEPLRE----Q**V**A**L**CSSG**R**V**V**R**VG**R**FLD**PAG**R**RR**LA**DE**LS**R**CL**

A3UIN0_1_88-169 **RDG**RRM**E**S**I**K**I**TREEIR**V**I**R**RFPTGHLI**QF**V**L**PSA**WTRV**IVEGEGEP--DVQTR**L**TAMG**KSL**I**VG**S**WLS**PR**ER**ES**LA**DA**IR**D**AL**

A1VTB4_1_93-172 L**HA**ADG**E**Q**I**SFSPEGQ**LA**I**E**VVRGLDTR**HYRM**NPA**WAHL**ERGGPRKD----R**L**W**L**CCSPL**RV**E**VA**TQ**LG**AG**EK**RRVERE**LK**Q**AL**

Q9A236_1_68-149 **R**A**A**RRV**E**R**I**Q**V**TAEAV**TV**S**R**EDEKGARTV**WT**SPTA**FTRV**GVEQPGEH--EVR**V**R**L**MIHR**KRL**T**LA**RA**LG**PDQ**R**LE**FG**AA**LQ**D**AI**

A2SKP6_1_95-173 **RHA**ADR**E**L**I**T**L**LP-GR**LV**V**E**HLNGGRIE**R**A**EF**VPD**WVRV**EPRDDDRS----L**I**E**L**SGQG**R**V**I**A**VG**R**Y**VRPEL**R**RA**LA**EEF**R**T**AL**

Q0BPC5_1_89-170 **R**A**V**RQT**E**I**V**T**L**SGTGP**GI**A**H**IDARGRSR**HMKI**RPG**W**L**RL**RLEECPGR--VPQ**L**I**L**SSRDG**E**FE**LA**RS**LG**ED**EK**RA**LA**ET**LQ**D**AL**

A4BQS4_1_74-156 **QRA**YDT**E**V**V**H**V**SE-SK**V**EI**D**KGRRRPER**HWSF**DRL**WSEV**ILAGPGHPWYPTR**L**A**V**RSRG**EQV**E**LG**R**FLA**DE**ER**ARV**A**GE**LR**R**WI**

A4SZC5_1_63-141 **RHA**LDC**E**T**I**E**I**DG-TR**LI**V**K**KFIGYKETI**YEF**NSR**WAKI**EPPIAGSK----TFH**I**IQSNL**RV**E**LG**Q**F**IRHEQQMA**LI**AS**IR**P**HL**

A0P3P2_1_83-165 **HSA**RTF**E**E**V**V**V**SRHEI**AI**R**K**VGPGKKYQ**EYRF**NPF**WVRL**TVDRIEDE-GVVK**V**T**L**QSRG**EKV**D**LG**N**FLN**PD**DR**TS**FA**GAMAN**AL**

A3NFM1_1_145-225 **RHA**VDYDC**V**A**L**TE-QR**L**EVIQCDGAQLR**RYDW**NPL**WV**A**V**DLDAAHAR--DPT**I**R**I**RHGS**ET**AL**VG**R**H**V**T**LA**RR**RHV**A**RE**LN**A**AL**

A1B3D8_1_80-159 G**TA**R--**E**VML**L**DRDRL**IL**T**R**SDPGRPDRI**WQT**NPY**WVRL**ALRQN-GP-VEDY**L**V**L**TDGK**REV**E**LG**A**FLA**PE**ER**MA**L**RND**L**ARR**L**

A5G1G0_1_85-166 **R**G**A**KAS**E**V**I**V**L**TDEAL**TI**T**R**TTPGGRRS**E**V**RL**EAG**W**L**RV**DVEEQAGT-N-PI**V**S**V**ANREA**R**QI**VG**MA**LG**DA**ER**RD**FA**DA**LK**A**AI**

A3VQB4_1_95-176 A**QG**RRH**E**R**L**I**L**TDDAL**WV**I**R**VLPSGHET**RWKL**TPA**FVRI**DIARPIEH--DSQ**L**C**L**RECG**KTL**V**IG**S**FLA**PK**ER**GEV**A**EA**LE**R**VL**

A3VHI7_1_90-172 **RDG**EIL**E**E**L**T**I**WTDRM**HL**S**R**TGPRRQHA**EWDA**NPH**WV**S**V**QVHKDGGP-VKHY**L**T**L**KGNG**REV**E**IG**S**FLS**ED**ER**PL**L**REE**LE**R**AL**

A6F2F8_1_72-154 **RRC**QRR**E**V**L**TFAP-EL**I**RL**E**KGLTRKEQ**EWEL**PRR**HTRV**WQDMPRHPWTPPK**L**H**L**QFRG**EEI**S**LA**P**FLN**ID**D**TEE**LV**AI**LE**R**H**G

Q2YZX7_1_124-204 **RRT**DVI**E**T**V**E**I**SP-RD**IT**V**H**RRELGREE**TK**V**F**PAY**WAHV**DFSGSPTQ--NGT**L**E**I**RSHG**E**A**I**E**IG**R**FLS**AS**EK**DRT**A**WK**LN**D**VL**

A6GLS2_1_97-175 **RHA**ADY**E**M**I**E**L**QP-NQ**LT**LVMADGTKLT**QLEW**SPQ**WAKL**SYNGKYKA----P**L**LFSHKGQ**QV**K**IG**K**F**I**A**EK**DK**SA**LH**RE**LK**A**AL**

A7HZ34_1_77-158 **K**A**A**RAH**E**T**V**Q**L**TDDEL**LV**R**R**VDAKGRARA**F**A**F**QPY**WVRL**ALRKEPDE--TTH**L**H**L**LSHG**RQL**E**VA**AA**LS**PP**ER**ES**FM**HA**LE**A**AL**

Q3J6Q7_1_74-156 **RRA**QHC**E**I**I**T**I**GQ-EE**I**EIFRGRETTGE**TWKF**HRY**WARV**RIELPPYAWHMSR**L**I**I**GSHG**HEV**E**IG**V**FLS**EE**ER**LR**LA**KE**LQ**A**V**C

B7L1X4_1_84-166 **RRG**RSF**E**E**V**A**I**SPLEV**FL**A**R**IDPRGVRR**EWRF**NPL**WTKL**SRIDDDEF-GLRT**L**T**L**TSRR**EHV**V**VA**RDA**S**PD**ER**AIV**A**DG**LT**R**AL**

Q28SZ7_1_86-168 **RD**MDLY**E**D**V**M**I**WDDLIR**V**E**R**HERRHALR**DWEA**NPY**WVR**MVLHAKGGP-VPNY**L**T**L**QGGP**REV**E**LG**A**FLT**PL**ER**VE**L**KQL**LD**RN**L**

A1TT19_1_80-163 **RHA**LDGDL**I**V**L**LDNGE**L**EICCLRGAQEQ**HYRF**PAA**WCRV**ECVPGRHRAERSG**L**C**I**ACGR**HRI**A**LG**A**W**G**S**PR**R**ADR**LA**GE**IR**A**A**A

Q2SMB1_1_74-155 **KRC**ASQ**E**V**I**L**I**TP-LE**VC**I**E**KGMAQPER**TWTF**PRW**YTRI**ILVEGGRN-GHIC**V**M**I**ACKG**EEV**E**IG**A**WLA**EG**DR**KA**LI**AT**LR**S**LV**

A3JQ23_1_89-171 L**DG**QLR**E**V**L**H**I**WEDRI**TL**T**H**IPRKGTAAF**WQA**NPY**WVKL**VKHDTGGR-VPEY**L**T**L**EGSG**K**I**V**E**LG**A**FLA**PE**ER**RE**LY**IY**LN**R**AL**

Q3SLX6_1_78-159 **ER**KDDY**E**R**L**T**I**DG-DR**VV**L**E**WRSRKREG**RREL**NRQ**WTRV**RCTCAAPG-RNCR**V**G**V**CCYG**RE**TL**VG**Q**YLS**DEA**R**LR**LA**AT**LR**SK**L**
